# Genome-wide physical activity interactions in adiposity ― A meta-analysis of 200,452 adults

**DOI:** 10.1371/journal.pgen.1006528

**Published:** 2017-04-27

**Authors:** Mariaelisa Graff, Robert A. Scott, Anne E. Justice, Kristin L. Young, Mary F. Feitosa, Llilda Barata, Thomas W. Winkler, Audrey Y. Chu, Anubha Mahajan, David Hadley, Luting Xue, Tsegaselassie Workalemahu, Nancy L. Heard-Costa, Marcel den Hoed, Tarunveer S. Ahluwalia, Qibin Qi, Julius S. Ngwa, Frida Renström, Lydia Quaye, John D. Eicher, James E. Hayes, Marilyn Cornelis, Zoltan Kutalik, Elise Lim, Jian’an Luan, Jennifer E. Huffman, Weihua Zhang, Wei Zhao, Paula J. Griffin, Toomas Haller, Shafqat Ahmad, Pedro M. Marques-Vidal, Stephanie Bien, Loic Yengo, Alexander Teumer, Albert Vernon Smith, Meena Kumari, Marie Neergaard Harder, Johanne Marie Justesen, Marcus E. Kleber, Mette Hollensted, Kurt Lohman, Natalia V. Rivera, John B. Whitfield, Jing Hua Zhao, Heather M. Stringham, Leo-Pekka Lyytikäinen, Charlotte Huppertz, Gonneke Willemsen, Wouter J. Peyrot, Ying Wu, Kati Kristiansson, Ayse Demirkan, Myriam Fornage, Maija Hassinen, Lawrence F. Bielak, Gemma Cadby, Toshiko Tanaka, Reedik Mägi, Peter J. van der Most, Anne U. Jackson, Jennifer L. Bragg-Gresham, Veronique Vitart, Jonathan Marten, Pau Navarro, Claire Bellis, Dorota Pasko, Åsa Johansson, Søren Snitker, Yu-Ching Cheng, Joel Eriksson, Unhee Lim, Mette Aadahl, Linda S. Adair, Najaf Amin, Beverley Balkau, Juha Auvinen, John Beilby, Richard N. Bergman, Sven Bergmann, Alain G. Bertoni, John Blangero, Amélie Bonnefond, Lori L. Bonnycastle, Judith B. Borja, Søren Brage, Fabio Busonero, Steve Buyske, Harry Campbell, Peter S. Chines, Francis S. Collins, Tanguy Corre, George Davey Smith, Graciela E. Delgado, Nicole Dueker, Marcus Dörr, Tapani Ebeling, Gudny Eiriksdottir, Tõnu Esko, Jessica D. Faul, Mao Fu, Kristine Færch, Christian Gieger, Sven Gläser, Jian Gong, Penny Gordon-Larsen, Harald Grallert, Tanja B. Grammer, Niels Grarup, Gerard van Grootheest, Kennet Harald, Nicholas D. Hastie, Aki S. Havulinna, Dena Hernandez, Lucia Hindorff, Lynne J. Hocking, Oddgeir L. Holmens, Christina Holzapfel, Jouke Jan Hottenga, Jie Huang, Tao Huang, Jennie Hui, Cornelia Huth, Nina Hutri-Kähönen, Alan L. James, John-Olov Jansson, Min A. Jhun, Markus Juonala, Leena Kinnunen, Heikki A. Koistinen, Ivana Kolcic, Pirjo Komulainen, Johanna Kuusisto, Kirsti Kvaløy, Mika Kähönen, Timo A. Lakka, Lenore J. Launer, Benjamin Lehne, Cecilia M. Lindgren, Mattias Lorentzon, Robert Luben, Michel Marre, Yuri Milaneschi, Keri L. Monda, Grant W. Montgomery, Marleen H. M. De Moor, Antonella Mulas, Martina Müller-Nurasyid, A. W. Musk, Reija Männikkö, Satu Männistö, Narisu Narisu, Matthias Nauck, Jennifer A. Nettleton, Ilja M. Nolte, Albertine J. Oldehinkel, Matthias Olden, Ken K. Ong, Sandosh Padmanabhan, Lavinia Paternoster, Jeremiah Perez, Markus Perola, Annette Peters, Ulrike Peters, Patricia A. Peyser, Inga Prokopenko, Hannu Puolijoki, Olli T. Raitakari, Tuomo Rankinen, Laura J. Rasmussen-Torvik, Rajesh Rawal, Paul M. Ridker, Lynda M. Rose, Igor Rudan, Cinzia Sarti, Mark A. Sarzynski, Kai Savonen, William R. Scott, Serena Sanna, Alan R. Shuldiner, Steve Sidney, Günther Silbernagel, Blair H. Smith, Jennifer A. Smith, Harold Snieder, Alena Stančáková, Barbara Sternfeld, Amy J. Swift, Tuija Tammelin, Sian-Tsung Tan, Barbara Thorand, Dorothée Thuillier, Liesbeth Vandenput, Henrik Vestergaard, Jana V. van Vliet-Ostaptchouk, Marie-Claude Vohl, Uwe Völker, Gérard Waeber, Mark Walker, Sarah Wild, Andrew Wong, Alan F. Wright, M. Carola Zillikens, Niha Zubair, Christopher A. Haiman, Loic Lemarchand, Ulf Gyllensten, Claes Ohlsson, Albert Hofman, Fernando Rivadeneira, André G. Uitterlinden, Louis Pérusse, James F. Wilson, Caroline Hayward, Ozren Polasek, Francesco Cucca, Kristian Hveem, Catharina A. Hartman, Anke Tönjes, Stefania Bandinelli, Lyle J. Palmer, Sharon L. R. Kardia, Rainer Rauramaa, Thorkild I. A. Sørensen, Jaakko Tuomilehto, Veikko Salomaa, Brenda W. J. H. Penninx, Eco J. C. de Geus, Dorret I. Boomsma, Terho Lehtimäki, Massimo Mangino, Markku Laakso, Claude Bouchard, Nicholas G. Martin, Diana Kuh, Yongmei Liu, Allan Linneberg, Winfried März, Konstantin Strauch, Mika Kivimäki, Tamara B. Harris, Vilmundur Gudnason, Henry Völzke, Lu Qi, Marjo-Riitta Järvelin, John C. Chambers, Jaspal S. Kooner, Philippe Froguel, Charles Kooperberg, Peter Vollenweider, Göran Hallmans, Torben Hansen, Oluf Pedersen, Andres Metspalu, Nicholas J. Wareham, Claudia Langenberg, David R. Weir, David J. Porteous, Eric Boerwinkle, Daniel I. Chasman, CHARGE Consortium, EPIC-InterAct Consortium, PAGE Consortium, Gonçalo R. Abecasis, Inês Barroso, Mark I. McCarthy, Timothy M. Frayling, Jeffrey R. O’Connell, Cornelia M. van Duijn, Michael Boehnke, Iris M. Heid, Karen L. Mohlke, David P. Strachan, Caroline S. Fox, Ching-Ti Liu, Joel N. Hirschhorn, Robert J. Klein, Andrew D. Johnson, Ingrid B. Borecki, Paul W. Franks, Kari E. North, L. Adrienne Cupples, Ruth J. F. Loos, Tuomas O. Kilpeläinen

**Affiliations:** 1 Department of Epidemiology, Gillings School of Global Public Health, University of North Carolina at Chapel Hill, Chapel Hill, North Carolina, United States of America; 2 MRC Epidemiology Unit, Institute of Metabolic Science, University of Cambridge, Cambridge, United Kingdom; 3 Carolina Population Center, University of North Carolina at Chapel Hill, Chapel Hill, North Carolina, United States of America; 4 Department of Genetics, Washington University School of Medicine, St. Louis, Missouri, United States of America; 5 Department of Genetic Epidemiology, University of Regensburg, Regensburg, Germany; 6 National Heart, Lung, and Blood Institute, Framingham Heart Study, Framingham, Massachusetts, United States of America; 7 Division of Preventive Medicine, Brigham and Women's Hospital, Boston, Massachusetts, United States of America; 8 Wellcome Trust Centre for Human Genetics, University of Oxford, Oxford, United Kingdom; 9 Division of Population Health Sciences and Education, St. George's, University of London, London, United Kingdom; 10 Department of Biostatistics, Boston University School of Public Health, Boston, Massachusetts, United States of America; 11 Department of Nutrition, Harvard T.H. Chan School of Public Health, Boston, Massachusetts, United States of America; 12 Department of Neurology, Boston University School of Medicine, Boston, Massachusetts, United States of America; 13 Department of Immunology, Genetics and Pathology and Science for Life Laboratory, Uppsala University, Uppsala, Sweden; 14 Novo Nordisk Foundation Center for Basic Metabolic Research, Section of Metabolic Genetics, Faculty of Health and Medical Sciences, University of Copenhagen, Copenhagen, Denmark; 15 Steno Diabetes Center, Gentofte, Denmark; 16 Department of Epidemiology and Population Health, Albert Einstein College of Medicine, Bronx, New York, United States of America; 17 Howard University, Department of Internal Medicine, Washington DC, United States of America; 18 Department of Clinical Sciences, Genetic and Molecular Epidemiology Unit, Lund University, Malmö, Sweden; 19 Department of Biobank Research, Umeå University, Umeå, Sweden; 20 Department of Twin Research and Genetic Epidemiology, King's College London, London, United Kingdom; 21 Population Sciences Branch, National Heart, Lung, and Blood Institute, National Institutes of Health, The Framingham Heart Study, Framingham, Massachusetts, United States of America; 22 Cell and Developmental Biology Graduate Program, Weill Cornell Graduate School of Medical Sciences, Cornell University, New York, New York, United States of America; 23 Icahn Institute for Genomics and Multiscale Biology, Icahn School of Medicine at Mount Sinai, New York, New York, United States of America; 24 Department of Preventive Medicine, Northwestern University Feinberg School of Medicine, Chicago, Illinois, United States of America; 25 Channing Division of Network Medicine, Department of Medicine, Brigham and Women's Hospital and Harvard Medical School, Boston, Massachusetts, United States of America; 26 Institute of Social and Preventive Medicine, Lausanne University Hospital, Lausanne, Switzerland; 27 Swiss Institute of Bioinformatics, Lausanne, Switzerland; 28 MRC Human Genetics Unit, Institute of Genetics and Molecular Medicine, University of Edinburgh, Western General Hospital, Edinburgh, United Kingdom; 29 Department of Epidemiology and Biostatistics, School of Public Health, Imperial College London, London, United Kingdom; 30 Department of Cardiology, Ealing Hospital HNS Trust, Middlesex, United Kingdom; 31 Department of Epidemiology, School of Public Health, University of Michigan, Ann Arbor, Michigan, United States of America; 32 Estonian Genome Center, University of Tartu, Tartu, Estonia; 33 Department of Internal Medicine, Internal Medicine, Lausanne University Hospital, Lausanne, Switzerland; 34 Division of Public Health Sciences, Fred Hutchinson Cancer Research Center, Seattle, Washington, United States of America; 35 University of Lille, CNRS, Institut Pasteur de Lille, UMR 8199 - EGID, Lille, France; 36 Institute for Community Medicine, University Medicine Greifswald, Greifswald, Germany; 37 DZHK (German Center for Cardiovascular Research), partner site Greifswald, Greifswald, Germany; 38 Icelandic Heart Association, Kopavogur, Iceland; 39 Faculty of Medicine, University of Iceland, Reykjavik, Iceland; 40 ISER, University of Essex, Colchester, Essex, United Kingdom; 41 Vth Department of Medicine, Medical Faculty Mannheim, Heidelberg University, Mannheim, Germany; 42 Institute of Nutrition, Friedrich Schiller University Jena, Jena, Germany; 43 Department of Biostatistical Sciences, Division of Public Health Sciences, Wake Forest School of Medicine, Winston-Salem, North Carolina, United States of America; 44 Karolinska Institutet, Respiratory Unit, Department of Medicine Solna, Stockholm, Sweden; 45 Genetic Epidemiology, QIMR Berghofer Medical Research Institute, Brisbane, Australia; 46 Center for Statistical Genetics, Department of Biostatistics, University of Michigan, Ann Arbor, Michigan, United States of America; 47 Department of Clinical Chemistry, Fimlab Laboratories, Tampere, Finland; 48 Department of Clinical Chemistry, University of Tampere School of Medicine, Tampere, Finland; 49 Department of Biological Psychology, Vrije Universiteit, Amsterdam, The Netherlands; 50 EMGO+ Institute, Vrije Universiteit & VU University Medical Center, Amsterdam, The Netherlands; 51 Department of Public and Occupational Health, VU University Medical Center, Amsterdam, The Netherlands; 52 Department of Psychiatry, EMGO Institute for Health and Care Research and Neuroscience Campus Amsterdam, VU University Medical Center/GGZ InGeest, Amsterdam, The Netherlands; 53 Department of Genetics, University of North Carolina, Chapel Hill, North Carolina, United States of America; 54 National Institute for Health and Welfare, Department of Health, Helsinki, Finland; 55 Institute for Molecular Medicine Finland, University of Helsinki, Helsinki, Finland; 56 Genetic Epidemiology Unit, Department of Epidemiology, Erasmus MC, Rotterdam, The Netherlands; 57 Department of Human Genetics, Leiden University Medical Center, Leiden, The Netherlands; 58 Institute of Molecular Medicine, University of Texas Health Science Center at Houston, Houston, Texas, United States of America; 59 Division of Epidemiology, Human Genetics, and Environmental Sciences, University of Texas Health Science Center at Houston, Houston, Texas, United States of America; 60 Kuopio Research Institute of Exercise Medicine, Kuopio, Finland; 61 Centre for Genetic Origins of Health and Disease, University of Western Australia, Crawley, Western Australia, Australia; 62 Translational Gerontology Branch, National Institute on Aging, Baltimore, Maryland, United States of America; 63 Department of Epidemiology, University of Groningen, University Medical Center Groningen, Groningen, The Netherlands; 64 Human Genetics, Genome Institute of Singapore, Agency for Science, Technology and Research of Singapore, Singapore; 65 Genomics Research Centre, Institute of Health and Biomedical Innovation, Queensland University of Technology, Brisbane, Queensland, Australia; 66 Genetics of Complex Traits, University of Exeter Medical School, University of Exeter, Exeter, United Kingdom; 67 Department of Immunology, Genetics and Pathology, Uppsala University, Uppsala, Sweden; 68 Division of Endocrinology, Diabetes, and Nutrition, University of Maryland School of Medicine, Baltimore, Maryland, United States of America; 69 Veterans Affairs Maryland Health Care System, University of Maryland, Baltimore, Maryland, United States of America; 70 Centre for Bone and Arthritis Research, Department of Internal Medicine and Clinical Nutrition, Institute of Medicine, Sahlgrenska Academy, University of Gothenburg, Gothenburg, Sweden; 71 Epidemiology Program, University of Hawaii Cancer Center, Honolulu, Hawaii, United States of America; 72 Research Centre for Prevention and Health, Glostrup University Hospital, Glostrup, Denmark; 73 Department of Public Health, Faculty of Health and Medical Sciences, University of Copenhagen, Copenhagen, Denmark; 74 Department of Nutrition, Gillings School of Global Public Health, University of North Carolina at Chapel Hill, Chapel Hill, North Carolina, United States of America; 75 INSERM U-1018, CESP, Renal and Cardiovascular Epidemiology, UVSQ-UPS, Villejuif, France; 76 Center for Life Course Health Research, Faculty of Medicine, University of Oulu, Oulu, Finland; 77 Unit of Primary Care, Oulu University Hospital, Oulu, Finland; 78 Busselton Population Medical Research Institute, Nedlands, Western Australia, Australia; 79 PathWest Laboratory Medicine of WA, Sir Charles Gairdner Hospital, Nedlands, Western Australia, Australia; 80 School of Pathology and Laboratory Medicine, The University of Western Australia, Crawley, Western Australia, Australia; 81 Diabetes and Obesity Research Institute, Cedars-Sinai Medical Center, Los Angeles, California, United States of America; 82 Department of Medical Genetics, University of Lausanne, Lausanne, Switzerland; 83 Department of Epidemiology and Prevention, Division of Public Health Sciences, Wake Forest School of Medicine, Winston-Salem, North Carolina, United States of America; 84 Department of Internal Medicine, Wake Forest School of Medicine, Winston-Salem, North Carolina, United States of America; 85 Texas Biomedical Research Institute, San Antonio, Texas, United States of America; 86 Medical Genomics and Metabolic Genetics Branch, National Human Genome Research Institute, NIH, Bethesda, Maryland, United States of America; 87 USC-Office of Population Studies Foundation, Inc., University of San Carlos, Cebu City, Philippines; 88 Department of Nutrition and Dietetics, University of San Carlos, Cebu City, Philippines; 89 Istituto di Ricerca Genetica e Biomedica (IRGB), Consiglio Nazionale Delle Ricerche (CNR), Cittadella Universitaria di Monserrato, Monserrato, Italy; 90 Department of Genetics, Rutgers University, Piscataway, New Jersey, United States of America; 91 Department of Statistics and Biostatistics, Rutgers University, Piscataway, New Jersey, United States of America; 92 Centre for Global Health Research, Usher Institute for Population Health Sciences and Informatics, Edinburgh, Scotland; 93 MRC Integrative Epidemiology Unit & School of Social and Community Medicine, University of Bristol, Bristol, United Kingdom; 94 University of Maryland School of Medicine, Department of Epidemiology & Public Health, Baltimore, Maryland, United States of America; 95 Department of Internal Medicine B, University Medicine Greifswald, Greifswald, Germany; 96 Department of Medicine, Oulu University Hospital, Oulu, Finland; 97 Institute of Clinical Medicine, Faculty of Medicine, University of Oulu, Oulu, Finland; 98 Division of Endocrinology, Boston Children's Hospital, Boston, Massachusetts, United States of America; 99 Department of Genetics, Harvard Medical School, Boston, Massachusetts, United States of America; 100 Broad Institute of the Massachusetts Institute of Technology and Harvard University, Cambridge, Massachusetts, United States of America; 101 Survey Research Center, Institute for Social Research, University of Michigan, Ann Arbor, Michigan, United States of America; 102 Research Unit of Molecular Epidemiology, Helmholtz Zentrum München - German Research Center for Environmental Health, Neuherberg, Germany; 103 Institute of Genetic Epidemiology, Helmholtz Zentrum München, German Research Center for Environmental Health, Neuherberg, Germany; 104 Institute of Epidemiology II, Helmholtz Zentrum München-German Research Center for Environmental Health, Neuherberg, Germany; 105 German Center for Diabetes Research (DZD), München-Neuherberg, Germany; 106 Laboratory of Neurogenetics, National Institute on Aging, Bethesda, Maryland, United States of America; 107 Division of Genomic Medicine, National Human Genome Research Institute, National Institutes of Health, Bethesda, Maryland, United States of America; 108 Musculoskeletal Research Programme, Division of Applied Medicine, University of Aberdeen, Foresterhill, Aberdeen, United Kingdom; 109 Generation Scotland, Centre for Genomic and Experimental Medicine, University of Edinburgh, Edinburgh, United Kingdom; 110 St. Olav Hospital, Trondheim University Hospital, Trondheim, Norway; 111 Institute for Nutritional Medicine, Klinikum Rechts der Isar, Technische Universität München, Munich, Germany; 112 NCA Institute, VU University & VU Medical Center, Amsterdam, The Netherlands; 113 Department of Human Genetics, Wellcome Trust Sanger Institute, Hinxton, Cambridge, United Kingdom; 114 School of Population Health, The University of Western Australia, Crawley, Western Australia, Australia; 115 Department of Pediatrics, Tampere University Hospital, Tampere, Finland; 116 Department of Pediatrics, University of Tampere School of Medicine, Tampere, Finland; 117 Department of Pulmonary Physiology and Sleep Medicine, Sir Charles Gairdner Hospital, Nedlands, Western Australia, Australia; 118 School of Medicine and Pharmacology, The University of Western Australia, Crawley, Western Australia, Australia; 119 Department of Physiology, Institute of Neuroscience and Physiology, Sahlgrenska Academy, University of Gothenburg, Gothenburg, Sweden; 120 Department of Medicine, University of Turku, Turku, Finland; 121 Division of Medicine, Turku University Hospital, Turku, Finland; 122 National Institute for Health and Welfare, Department of Health, Helsinki, Finland; 123 Department of Medicine and Abdominal Center: Endocrinology, University of Helsinki and Helsinki University Central Hospital, Helsinki, Finland; 124 Minerva Foundation Institute for Medical Research, Helsinki, Finland; 125 Department of Public Health, Faculty of Medicine, University of Split, Split, Croatia; 126 Department of Medicine, University of Eastern Finland and Kuopio University Hospital, Kuopio, Finland; 127 HUNT Research Centre, Department of Public Health and General Practice, Norwegian University of Science and Technology, Levanger, Norway; 128 Department of Clinical Physiology, Tampere University Hospital, Tampere, Finland; 129 Department of Clinical Physiology, University of Tampere School of Medicine, Tampere, Finland; 130 Institute of Biomedicine, Physiology, University of Eastern Finland, Kuopio Campus, Finland; 131 Neuroepidemiology Section, National Institute on Aging, National Institutes of Health, Bethesda, Maryland, United States of America; 132 Program in Medical and Population Genetics, Broad Institute, Cambridge, Massachusetts, United States of America; 133 The Big Data Institute, University of Oxford, Oxford, United Kingdom; 134 Geriatric Medicine, Sahlgrenska University Hospital, Mölndal, Sweden; 135 Department of Public Health and Primary Care, University of Cambridge, Cambridge, United Kingdom; 136 INSERM U-1138, Équipe 2: Pathophysiology and Therapeutics of Vascular and Renal diseases Related to Diabetes, Centre de Recherche des Cordeliers, Paris, France; 137 Department of Endocrinology, Diabetology, Nutrition, and Metabolic Diseases, Bichat Claude Bernard Hospital, Paris, France; 138 Center for Observational Research, Amgen Inc., Thousand Oaks, California, United States of America; 139 Section of Clinical Child and Family Studies, Department of Educational and Family Studies, Vrije Universiteit, Amsterdam, The Netherlands; 140 Dipartimento di Scienze Biomediche, Università degli Studi di Sassari, Sassari, Italy; 141 Department of Medicine I, Ludwig-Maximilians-Universität, Munich, Germany; 142 DZHK (German Centre for Cardiovascular Research), partner site Munich Heart Alliance, Munich, Germany; 143 Department of Respiratory Medicine, Sir Charles Gairdner Hospital, Nedlands, Western Australia, Australia; 144 Institute of Clinical Chemistry and Laboratory Medicine, University Medicine Greifswald, Greifswald, Germany; 145 Interdisciplinary Center Psychopathology and Emotion Regulation (ICPE), University of Groningen, University Medical Center Groningen, Groningen, The Netherlands; 146 Institute of Cardiovascular and Medical Sciences, BHF Glasgow Cardiovascular Research Centre, University of Glasgow, Glasgow, United Kingdom; 147 University of Tartu, Estonian Genome Centre, Tartu, Estonia; 148 Genomics of Common Disease, Imperial College London, London, United Kingdom; 149 South Ostrobothnia Central Hospital, Seinäjoki, Finland; 150 Department of Clinical Physiology and Nuclear Medicine, Turku University Hospital, Turku, Finland; 151 Research Centre of Applied and Preventive Cardiovascular Medicine, University of Turku, Turku, Finland; 152 Human Genomics Laboratory, Pennington Biomedical Research Center, Baton Rouge, Louisiana, United States of America; 153 Harvard Medical School, Boston, Massachusetts, United States of America; 154 Social Services and Health Care Department, City of Helsinki, Helsinki, Finland; 155 Division of Research, Kaiser Permanente Northern California, Oakland, California, United States of America; 156 Division of Angiology, Department of Internal Medicine, Medical University Graz, Austria; 157 School of Medicine, University of Dundee, Ninewells Hospital and Medical School, Dundee, Scotland; 158 LIKES Research Center for Sport and Health Sciences, Jyväskylä, Finland; 159 National Heart and Lung Institute, Imperial College London, United Kingdom; 160 Department of Endocrinology, University of Groningen, University Medical Center Groningen, Groningen, The Netherlands; 161 Institute of Nutrition and Functional Foods, Quebec, Canada; 162 School of Nutrition, Laval University, Quebec, Canada; 163 Interfaculty Institute for Genetics and Functional Genomics, University Medicine Greifswald, Germany; 164 Institute of Cellular Medicine, Newcastle University, Newcastle upon Tyne, United Kingdom; 165 Centre for Population Health Sciences, Usher Institute for Population Health Sciences and Informatics, Teviot Place, Edinburgh, Scotland; 166 MRC Unit for Lifelong Health and Ageing at UCL, London, United Kingdom; 167 Department of Internal Medicine, Erasmus MC, Rotterdam, The Netherlands; 168 Department of Preventive Medicine, Norris Comprehensive Cancer Center, Keck School of Medicine, University of Southern California, Los Angeles, California, United States of America; 169 Department of Epidemiology, Erasmus MC, Rotterdam, The Netherlands; 170 Netherlands Consortium for Healthy Aging, Leiden University Medical Center, Leiden, The Netherlands; 171 Department of Kinesiology, Laval University, Quebec, Canada; 172 Department of Psychiatry, University of Groningen, University Medical Center Groningen, Groningen, The Netherlands; 173 University of Leipzig, Medical Department, Leipzig, Germany; 174 Geriatric Unit, Azienda Sanitaria Firenze, Florence, Italy; 175 School of Public Health, University of Adelaide, Adelaide, South Australia, Australia; 176 Department of Clinical Physiology and Nuclear Medicine, Kuopio University Hospital, Kuopio, Finland; 177 Department of Clinical Epidemiology, Bispebjerg and Frederiksberg Hospitals, The Capital Region, Copenhagen, Denmark; 178 Centre for Vascular Prevention, Danube-University Krems, Krems, Austria; 179 Diabetes Research Group, King Abdulaziz University, Jeddah, Saudi Arabia; 180 National Institute for Health Research Biomedical Research Centre at Guy's and St. Thomas' Foundation Trust, London, United Kingdom; 181 Department of Clinical Experimental Research, Rigshospitalet, Glostrup, Denmark; 182 Department of Clinical Medicine, Faculty of Health and Medical Sciences, University of Copenhagen, Copenhagen, Denmark; 183 Synlab Academy, Synlab Services LLC, Mannheim, Germany; 184 Clinical Institute of Medical and Chemical Laboratory Diagnostics, Medical University of Graz, Graz, Austria; 185 Institute of Medical Informatics, Biometry and Epidemiology, Chair of Genetic Epidemiology, Ludwig-Maximilians-Universität, Munich, Germany; 186 Department of Epidemiology and Public Health, University College London, London, United Kingdom; 187 Laboratory of Epidemiology and Population Science, National Institute on Aging, Bethesda, Maryland, United States of America; 188 Biocenter Oulu, University of Oulu, Oulu, Finland; 189 MRC-PHE Centre for Environment and Health, Imperial College London, London, United Kingdom; 190 Imperial College Healthcare NHS Trust, London, United Kingdom; 191 Hammersmith Hospital, London, United Kingdom; 192 Centre for Genomic and Experimental Medicine, Institute of Genetics and Molecular Medicine, University of Edinburgh, Edinburgh, United Kingdom; 193 Wellcome Trust Sanger Institute, Hinxton, United Kingdom; 194 NIHR Cambridge Biomedical Research Centre, Institute of Metabolic Science, Addenbrooke’s Hospital, Cambridge, United Kingdom; 195 The University of Cambridge Metabolic Research Laboratories, Wellcome Trust-MRC Institute of Metabolic Science, Cambridge, United Kingdom; 196 Oxford Centre for Diabetes, Endocrinology and Metabolism, University of Oxford, Churchill Hospital, Oxford, United Kingdom; 197 Oxford NIHR Biomedical Research Centre, Oxford, United Kingdom; 198 Center of Medical Systems Biology, Leiden, The Netherlands; 199 Population Health Research Institute, St. George's University of London, London, United Kingdom; 200 Divisions of Endocrinology and Genetics and Center for Basic and Translational Obesity Research, Boston Children's Hospital, Boston, Massachusetts, United States of America; 201 Department of Public Health & Clinical Medicine, Umeå University, Umeå, Sweden; 202 Carolina Center for Genome Sciences, Gillings School of Global Public Health, University of North Carolina at Chapel Hill, Chapel Hill, North Carolina, United States of America; 203 Genetics of Obesity and Related Metabolic Traits Program, Charles Bronfman Institute for Personalized Medicine, Icahn School of Medicine at Mount Sinai, New York, New York, United States of America; 204 The Mindich Child Health and Development Institute, Icahn School of Medicine at Mount Sinai, New York, New York, United States of America; 205 The Department of Preventive Medicine, The Icahn School of Medicine at Mount Sinai, New York, New York, United States of America; Vanderbilt University, UNITED STATES

## Abstract

Physical activity (PA) may modify the genetic effects that give rise to increased risk of obesity. To identify adiposity loci whose effects are modified by PA, we performed genome-wide interaction meta-analyses of BMI and BMI-adjusted waist circumference and waist-hip ratio from up to 200,452 adults of European (n = 180,423) or other ancestry (n = 20,029). We standardized PA by categorizing it into a dichotomous variable where, on average, 23% of participants were categorized as inactive and 77% as physically active. While we replicate the interaction with PA for the strongest known obesity-risk locus in the *FTO* gene, of which the effect is attenuated by ~30% in physically active individuals compared to inactive individuals, we do not identify additional loci that are sensitive to PA. In additional genome-wide meta-analyses adjusting for PA and interaction with PA, we identify 11 novel adiposity loci, suggesting that accounting for PA or other environmental factors that contribute to variation in adiposity may facilitate gene discovery.

## Author summary

Decline in daily physical activity is thought to be a key contributor to the global obesity epidemic. However, the impact of sedentariness on adiposity may be in part determined by a person’s genetic constitution. The specific genetic variants that are sensitive to physical activity and regulate adiposity remain largely unknown. Here, we aimed to identify genetic variants whose effects on adiposity are modified by physical activity by examining ~2.5 million genetic variants in up to 200,452 individuals. We also tested whether adjusting for physical activity as a covariate could lead to the identification of novel adiposity variants. We find robust evidence of interaction with physical activity for the strongest known obesity risk-locus in the *FTO* gene, of which the body mass index-increasing effect is attenuated by ~30% in physically active individuals compared to inactive individuals. Our analyses indicate that other similar gene-physical activity interactions may exist, but better measurement of physical activity, larger sample sizes, and/or improved analytical methods will be required to identify them. Adjusting for physical activity, we identify 11 novel adiposity variants, suggesting that accounting for physical activity or other environmental factors that contribute to variation in adiposity may facilitate gene discovery.

## Introduction

In recent decades, we have witnessed a global obesity epidemic that may be driven by changes in lifestyle such as easier access to energy-dense foods and decreased physical activity (PA) [1]. However, not everyone becomes obese in obesogenic environments. Twin studies suggest that changes in body weight in response to lifestyle interventions are in part determined by a person’s genetic constitution [2–4]. Nevertheless, the genes that are sensitive to environmental influences remain largely unknown.

Previous studies suggest that genetic susceptibility to obesity, assessed by a genetic risk score for BMI, may be attenuated by PA [5, 6]. A large-scale meta-analysis of the *FTO* obesity locus in 218,166 adults showed that being physically active attenuates the BMI-increasing effect of this locus by ~30% [7]. While these findings suggest that *FTO*, and potentially other previously established BMI loci, may interact with PA, it has been hypothesized that loci showing the strongest main effect associations in genome-wide association studies (GWAS) may be the least sensitive to environmental and lifestyle influences, and may therefore not make the best candidates for interactions [8]. Yet no genome-wide search for novel loci exhibiting SNP×PA interaction has been performed. A genome-wide meta-analysis of genotype-dependent phenotypic variance of BMI, a marker of sensitivity to environmental exposures, in ~170,000 participants identified *FTO*, but did not show robust evidence of environmental sensitivity for other loci [9]. Recent genome-wide meta-analyses of adiposity traits in >320,000 individuals uncovered loci interacting with age and sex, but also suggested that very large sample sizes are required for interaction studies to be successful [10].

Here, we report results from a large-scale genome-wide meta-analysis of SNP×PA interactions in adiposity in up to 200,452 adults. As part of these interaction analyses, we also examine whether adjusting for PA or jointly testing for SNP’s main effect and interaction with PA may identify novel adiposity loci.

## Results

### Identification of loci interacting with PA

We performed meta-analyses of results from 60 studies, including up to 180,423 adults of European descent and 20,029 adults of other ancestries to assess interactions between ~2.5 million genotyped or HapMap-imputed SNPs and PA on BMI and BMI-adjusted waist circumference (WC_adjBMI_) and waist-hip ratio (WHR_adjBMI_) (S1–S5 Tables). Similar to a previous meta-analysis of the interaction between *FTO* and PA [7], we standardized PA by categorizing it into a dichotomous variable where on average ~23% of participants were categorized as inactive and ~77% as physically active (see Methods and S6 Table). On average, inactive individuals had 0.99 kg/m^2^ higher BMI, 3.46 cm higher WC, and 0.018 higher WHR than active individuals (S4 and S5 Tables).

Each study first performed genome-wide association analyses for each SNP’s effect on BMI in the inactive and active groups separately. Corresponding summary statistics from each cohort were subsequently meta-analyzed, and the SNP×PA interaction effect was estimated by calculating the difference in the SNP’s effect between the inactive and active groups. To identify sex-specific SNP×PA interactions, we performed the meta-analyses separately in men and women, as well as in the combined sample. In addition, we carried out meta-analyses in European-ancestry studies only and in European and other-ancestry studies combined.

**Fig 1:**
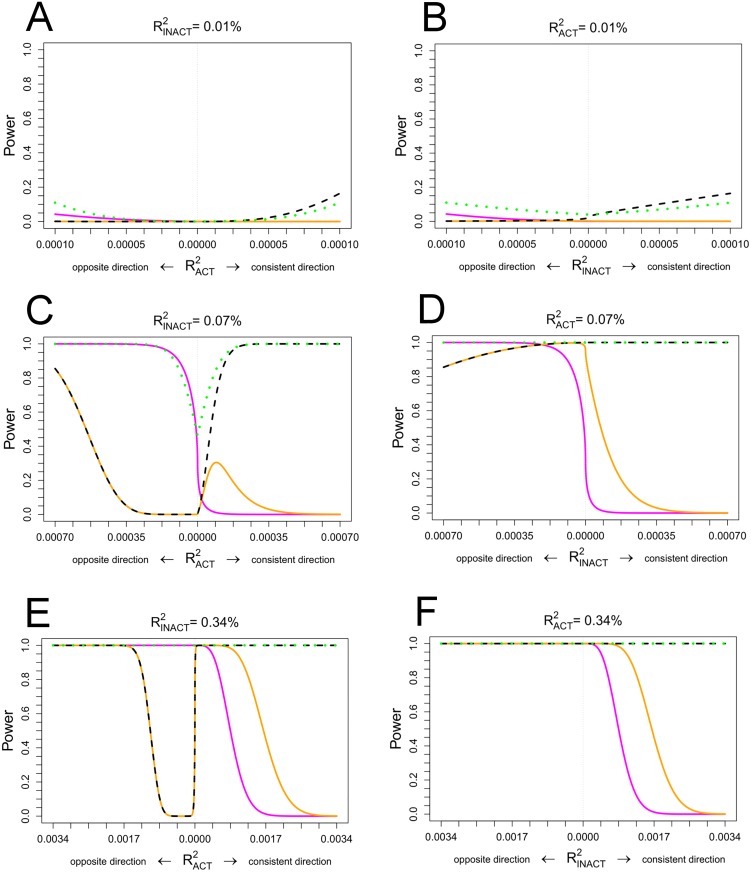
Power to identify PA-adjusted main, joint or GxPA interaction effects in 200,000 individuals (45,000 inactive, 155,000 active). The plots compare power to identify genome-wide significant main effects (P_adjPA_<5x10^-8^, dashed black), joint effects (P_JOINT_<5x10^-8^, dotted green) or GxPA interaction effects (P_INT_<5x10^-8^, solid magenta) as well as the power to identify Bonferroni-corrected interaction effects (P_INT_<0.05/number of loci, solid orange) for the SNPs that reached a genome-wide significant PA-adjusted main effect association (P_adjPA_<5x10^-8^). The power computations were based on analytical power formulae provided elsewhere [50] and were conducted a-priori based on various types of known realistic BMI effect sizes [51]. **Panels A, C, E**: Assuming an effect in inactive individuals similar to a small ($R_{INACT}^{2}=0.01\%$, comparable to the known BMI effect of the *NUDT3* locus), medium ($R_{INACT}^{2}=0.07\%$, comparable to the known BMI effect of the *BDNF* locus) and large ($R_{INACT}^{2}=0.34\%$, comparable to the known BMI effect of the *FTO* locus) realistic effect on BMI and for various effects in physically active individuals (varied on the x axis); **Panels B,D,F**: Assuming an effect in physically active individuals similar to the small, medium and large realistic effects of the *NUDT3*, *BDNF and FTO* loci on BMI and for various effects in inactive individuals (varied on x axis).

We used two approaches to identify loci whose effects are modified by PA. In the first approach, we searched for genome-wide significant SNP×PA interaction effects (P_INT_<5x10^-8^). As shown in Fig 1, this approach yielded the highest power to identify *cross-over* interaction effects where the SNP’s effect is directionally opposite between the inactive and active groups. However, this approach has low power to identify interaction effects where the SNP’s effect is directionally concordant between the inactive and active groups (Fig 1). We identified a genome-wide significant interaction between rs986732 in *cadherin 12 (CDH12)* and PA on BMI in European-ancestry studies (beta_INT_ = -0.076 SD/allele, P_INT_ = 3.1x10^-8^, n = 134,767) (S7 Table). The interaction effect was directionally consistent but did not replicate in an independent sample of 31,097 individuals (beta_INT_ = -0.019 SD/allele, P_INT_ = 0.52), and the pooled association P value for the discovery and replication stages combined did not reach genome-wide significance (N_TOTAL_ = 165,864; P_INT-TOTAL_ = 3x10^-7^) (S1 Fig). No loci showed genome-wide significant interactions with PA on WC_adjBMI_ or WHR_adjBMI_. *CDH12* encodes an integral membrane protein mediating calcium-dependent cell-cell adhesion in the brain, where it may play a role in neurogenesis [11]. While *CDH12* rs4701252 and rs268972 SNPs have shown suggestive associations with waist circumference (P = 2x10^-6^) and BMI (P = 5x10^-5^) in previous GWAS [12, 13], the SNPs are not in LD with rs986732 (r^2^<0.1).

In our second approach, we tested interaction for loci showing a genome-wide significant main effect on BMI, WC_adjBMI_ or WHR_adjBMI_ (S7–S12 Tables). We adjusted the significance threshold for SNP×PA interaction by Bonferroni correction (P = 0.05/number of SNPs tested). As shown in Fig 1, this approach enhanced our power to identify interaction effects where there is a difference in the magnitude of the SNP’s effect between inactive and active groups when the SNP’s effect is directionally concordant between the groups. We identified a significant SNP×PA interaction of the *FTO* rs9941349 SNP on BMI in the meta-analysis of European-ancestry individuals; the BMI-increasing effect was 33% smaller in active individuals (beta_ACTIVE_ = 0.072 SD/allele) than in inactive individuals (beta_INACTIVE_ = 0.106 SD/allele, P_INT_ = 4x10^-5^). The rs9941349 SNP is in strong LD (r^2^ = 0.87) with *FTO* rs9939609 for which interaction with PA has been previously established in a meta-analysis of 218,166 adults [7]. We identified no loci interacting with PA for WC_adjBMI_ or WHR_adjBMI_.

In a previously published meta-analysis [7], the *FTO* locus showed a geographic difference for the interaction effect where the interaction was more pronounced in studies from North America than in those from Europe. To test for geographic differences in the present study, we performed additional meta-analyses for the *FTO* rs9941349 SNP, stratified by geographic origin (North America vs. Europe). While the interaction effect was more pronounced in studies from North America (beta_INT_ = 0.052 SD/allele, P = 5x10^-4^, N = 63,896) than in those from Europe (beta_INT_ = 0.028 SD/allele, P = 0.006, N = 109,806), we did not find a statistically significant difference between the regions (P = 0.14).

#### Explained phenotypic variance in inactive and active individuals

We tested whether the variance explained by ~1.1 million common variants (MAF≥1%) differed between the inactive and active groups for BMI, WC_adjBMI_, and WHR_adjBMI_ [14]. In the physically active individuals, the variants explained ~20% less of variance in BMI than in inactive individuals (12.4% vs. 15.7%, respectively; P_difference_ = 0.046), suggesting that PA may reduce the impact of genetic predisposition to adiposity overall. There was no significant difference in the variance explained between active and inactive groups for WC_adjBMI_ (8.6% for active, 9.3% for inactive; P_difference_ = 0.70) or WHR_adjBMI_ (6.9% for active, 8.0% for inactive; P_difference_ = 0.59).

To further investigate differences in explained variance between the inactive and active groups, we calculated variance explained by subsets of SNPs selected based on significance thresholds (ranging from P = 5x10^-8^ to P = 0.05) of PA-adjusted SNP association with BMI, WC_adjBMI_ or WHR_adjBMI_ [15] (S13 Table). We found 17–26% smaller explained variance for BMI in the active group than in the inactive group at all P value thresholds (S13 Table).

### Identification of novel loci when adjusting for PA or when jointly testing for SNP main effect and interaction with PA

Physical activity contributes to variation in BMI, WC_adjBMI_, and WHR_adjBMI_, hence, adjusting for PA as a covariate may enhance power to identify novel adiposity loci. To that extent, each study performed genome-wide analyses for association with BMI, WC_adjBMI_, and WHR_adjBMI_ while adjusting for PA. Subsequently, we performed meta-analyses of the study-specific results. We discovered 10 genome-wide significant loci (2 for BMI, 1 for WC_adjBMI_, 7 for WHR_adjBMI_) that have not been reported in previous GWAS of adiposity traits (Table 1, S2–S4 Figs).

**Table 1:** Novel loci achieving genome-wide significance (P<5x10^-8^) in meta-analyses for PA-adjusted SNP main effect (P_adjPA_) or the joint test of SNP main effect and SNP-PA interaction (P_joint_).

Trait	Marker	Nearest Gene	Chr	Pos (hg19)	Trait increasing/decreasing allele	Trait increasing allele’s frequency	Analysis	N_adjPA_	Beta_adjPA_	SE_adjPA_	P_adjPA_	P_int_	P_joint_
**Novel loci achieving genome-wide significance in European-ancestry meta-analyses**													
BMI	rs1720825	*MRAS*	3	138,108,083	A/G	0.20	Overall	178833	0.026	0.0047	**2.98E-08**	1.62E-01	**3.67E-08**
							Women	102854	0.0281	0.006	2.84E-06	7.27E-02	3.35E-06
							Men	47544	0.024	0.0069	4.91E-04	9.95E-01	1.30E-02
BMI	rs1934100	*ELAVL2*	9	23,234,308	A/T	0.68	Overall	140811	0.0179	0.0049	2.43E-04	3.99E-02	2.15E-04
							Women	85142	0.0048	0.006	4.18E-01	9.89E-01	7.37E-01
							Men	41958	0.0377	0.0074	3.18E-07	8.84E-04	**3.70E-08**
WC_adjBMI_	rs7176527	*ZSCAN2*	15	85,140,794	C/T	0.81	Overall	130413	0.0317	0.0054	**5.98E-09**	1.79E-01	**2.80E-08**
							Women	77349	0.0303	0.007	1.37E-05	9.36E-01	1.28E-04
							Men	52918	0.0342	0.0084	4.55E-05	3.23E-02	7.50E-06
WHR_adjBMI_	rs4650943	*PAPPA2*	1	176,414,781	A/G	0.53	Overall	113963	0.0267	0.0048	**2.34E-08**	1.77E-01	5.76E-08
							Women	69016	0.0301	0.006	4.66E-07	7.79E-03	1.57E-07
							Men	44430	0.0212	0.0073	3.55E-03	2.73E-01	5.64E-03
WHR_adjBMI_	rs2300481	*MEIS1*	2	66,782,467	T/C	0.39	Overall	110881	0.0267	0.0048	**2.41E-08**	5.80E-01	**3.93E-08**
							Women	66519	0.0288	0.0059	1.19E-06	4.71E-01	1.47E-06
							Men	43845	0.0258	0.0073	4.14E-04	1.00E+00	2.82E-03
WHR_adjBMI_	rs167025	*ARHGEF28*	5	73,433,308	A/G	0.33	Overall	117603	0.0179	0.0048	2.13E-04	8.01E-01	4.64E-04
							Women	70494	0.0023	0.006	7.01E-01	4.50E-01	7.32E-01
							Men	46591	0.0427	0.0074	**6.24E-09**	1.34E-01	**3.73E-09**
WHR_adjBMI_	rs3094013	*HCP5*	6	31,434,366	G/A	0.87	Overall	149338	0.0269	0.0061	1.06E-05	4.98E-01	6.93E-05
							Women	84538	0.0104	0.0078	1.82E-01	4.50E-01	3.78E-01
							Men	64138	0.0494	0.009	**4.51E-08**	8.91E-01	7.87E-07
WHR_adjBMI_	rs6976930	*BAZ1B*	7	72,885,810	G/A	0.81	Overall	145913	0.0294	0.0051	**1.03E-08**	5.28E-01	**1.87E-08**
							Women	83184	0.0326	0.0066	7.70E-07	7.00E-01	2.02E-06
							Men	62149	0.0254	0.0075	7.69E-04	5.93E-01	3.10E-03
WHR_adjBMI_	rs10786152	*PLCE1*	10	95,893,514	A/G	0.52	Overall	147123	0.0224	0.004	**1.79E-08**	8.76E-02	**1.44E-08**
							Women	83884	0.0192	0.0051	1.56E-04	5.81E-02	1.41E-04
							Men	62722	0.0255	0.0058	1.32E-05	6.38E-01	5.89E-05
WHR_adjBMI_	rs889512	*CTRB2*	16	75,242,012	C/G	0.88	Overall	117417	0.031	0.0074	2.70E-05	4.26E-01	1.13E-04
							Women	70315	0.0506	0.0091	**2.87E-08**	9.96E-02	1.09E-07
							Men	46440	-0.0022	0.0114	8.50E-01	5.06E-01	7.80E-01
**Novel loci achieving genome-wide significance in all-ancestry meta-analyses**													
BMI	rs754635	*CCK*	3	42,305,131	G/C	0.87	Overall	151282	0.0356	0.0062	**1.07E-08**	1.21E-01	3.28E-07
							Women	91241	0.026	0.0079	9.66E-04	1.25E-01	8.69E-04
							Men	62741	0.0486	0.0093	1.61E-07	2.98E-01	3.68E-06

Chr: chromosome; Pos(hg19): position based on human assembly 19; N_adjPA_, Beta_adjPA_, SE_adjPA_, or P_adjPA_: sample size, effect size, standard error, or P value, respectively, in the physical activity adjusted SNP main effect model; PA: physical activity; WC_adjBMI_: BMI-adjusted waist circumference; WHR_adjBMI_: BMI-adjusted waist-hip ratio; P_int_: P value for SNP-PA interaction; P_joint_: P value for the joint test of SNP main effect and SNP-PA interaction.

To establish whether additionally accounting for SNP×PA interactions would identify novel loci, we calculated the joint significance of PA-adjusted SNP main effect and SNP×PA interaction using the method of Aschard et al [16]. As illustrated in Fig 1, the joint test enhanced our power to identify loci where the SNP shows simultaneously a main effect and an interaction effect. We identified a novel BMI locus near *ELAVL2* in men (P_JOINT_ = 4x10^-8^), which also showed suggestive evidence of interaction with PA (P_INT_ = 9x10^-4^); the effect of the BMI-increasing allele was attenuated by 71% in active as compared to inactive individuals (beta_INACTIVE_ = 0.087 SD/allele, beta_ACTIVE_ = 0.025 SD/allele) (Table 1, S2–S4 Figs).

To evaluate the effect of PA adjustment on the results for the 11 novel loci, we performed a look-up in published GIANT consortium meta-analyses for BMI, WC_adjBMI_, and WHR_adjBMI_ that did not adjust for PA [17, 18] (S22 Table). All 11 loci showed a consistent direction of effect between the present PA-adjusted and the previously published PA-unadjusted results, but the PA-unadjusted associations were less pronounced despite up to 40% greater sample size, suggesting that adjustment for PA may have increased our power to identify these loci.

The biological relevance of putative candidate genes in the novel loci, based on our thorough searches of the literature, GWAS catalog look-ups, and analyses of eQTL enrichment and overlap with functional regulatory elements, are described in Tables 2 and 3. As the novel loci were identified in a PA-adjusted model, where adjusting for PA may have contributed to their identification, we examined whether the lead SNPs in these loci are associated with the level of PA. More specifically, we performed look-ups in GWAS analyses for the levels of moderate-to-vigorous intensity leisure-time PA (n = 80,035), TV-viewing time (n = 28,752), and sedentary behavior at work (n = 59,381) or during transportation (n = 15,152) [personal communication with Marcel den Hoed, Marilyn Cornelis, and Ruth Loos]. However, we did not find significant associations when correcting for the number of loci that were examined (P>0.005) (S16 Table).

**Table 2:** Genes of biological interest within 500 kb of lead SNPs associated with BMI.

***CCK (rs754635)*:** The lead SNP is located in intron 1 of the *CCK* gene that encodes cholecystokinin, a gastrointestinal peptide that stimulates the digestion of fat and protein in the small intestine by inhibiting gastric emptying, inducing the release of pancreatic enzymes, increasing production of hepatic bile, and causing contraction of the gallbladder. Cholecystokinin induces satiety and reduces the amount of food consumed when administered prior to a meal [52, 53]. In a candidate gene study, four common variants in *CCK* were associated with increased meal size [54], but the variants are not in LD with rs754635 (r^2^<0.1). A GWAS of BMI in 62,246 individuals of East Asian ancestry showed a suggestive association (P = 2x10^-7^) for the rs4377469 SNP in high LD with our lead SNP (r^2^ = 0.7) [55].

***ELAVL2 (rs1934100)*:** The lead SNP showed an association with BMI only in men (Table 1). The only nearby gene *ELAVL2* (455 kb away) is a conserved neuron-specific RNA-binding protein involved in stabilization or enhanced translation of specific mRNAs with AU-rich elements in the 3’-untranslated region [56]. While *ELAVL2* is implicated in neuronal differentiation [56], potential mechanisms linking this function to obesity remain unclear.

***MRAS (rs1720825)*:** The lead SNP is an intronic variant in *MRAS*. The *MRAS* rs1199333 SNP, in high LD with rs1720825 (r^2^ = 0.85), has shown suggestive association with typical sporadic amyotrophic lateral sclerosis in a Chinese Han population (P = 4x10^-6^, S14 Table). Other *MRAS* SNPs have been associated with risk of coronary artery disease [57] but they are not in LD with rs1720825 (r^2^<0.06). *MRAS* encodes a member of the membrane-associated Ras small GTPase protein family that function as signal transducers in multiple processes of cell growth and differentiation and are involved in energy expenditure, adipogenesis, muscle differentiation, insulin signaling and glucose metabolism [58–60]. Mice with *Mras* knockout develop a severe obesity phenotype [61]. The SNP rs1199334, in high LD with our lead SNP rs1720825 (r^2^ = 0.90), has been identified as the SNP most strongly associated with the *cis*-expression of centrosomal protein 70kDa (*CEP70*) in subcutaneous adipose tissue (P = 2x10^-7^) (S15 Table). *CEP70* encodes a centrosomal protein that is critical for the regulation of mitotic spindle assembly, playing an essential role in cell cycle progression [62].

**Table 3:** Genes of biological interest within 500 kb of lead SNPs associated with WC_adjBMI_ or WHR_adjBMI_.

***ZSCAN2 (rs7176527)*:** Twenty two genes lie within 500kb of the WC_adjBMI_-associated lead SNP (S3 Fig). The nearest gene, *ZSCAN2*, contains several copies of a zinc finger motif commonly found in transcriptional regulatory proteins. The rs7176527 SNP is in LD (r^2^>0.80) with five SNPs (rs3762168, rs2762169, rs12594450, rs72630460, and rs16974951) that are enhancers in multiple tissues in the data from Roadmap Epigenomics Consortium [22]. The rs7176527 SNP is a *cis*-eQTL for the putative transcriptional regulator *SCAND2* [63] in the intestine, prefrontal cortex, and lymphocytes (S15 Table).

***PAPPA2 (rs4650943)*:** Seven genes lie within 500kb of the lead SNP (S3 Fig). The nearest gene, *PAPPA2*, is 18 kb upstream of rs4650943 and codes for a protease that locally regulates insulin-like growth factor availability through cleavage of IGF binding protein 5, most commonly found in bone tissue. In murine models, the PAPP-A2 protein has been shown to influence overall body size and bone growth, but not glucose metabolism or adiposity [64–66].

***MEIS1 (rs2300481)*:** The only gene within 500 kb of the lead SNP is *MEIS1* encoding a homeobox protein that plays an important role in normal organismal growth and development. Two variants in high LD with the lead SNP (r^2^ = 0.95) have been identified for association with PR interval of the heart (S14 Table). Another variant, in low LD with rs2300481 (r^2^ = 0.25), has been associated with restless leg syndrome [67]–a sleeping disorder that may cause weight gain [68].

***ARHGEF28 (rs167025)*:** The lead SNP showed an association with WHR_adjBMI_ in men only (Table 1). There are two protein-coding genes within 500kb of rs167025. The nearest gene is *ARHGEF28*, 195 kb downstream, encoding Rho guanine nucleotide exchange factor 28. This exchange factor has been shown to destabilize low molecular weight neurofilament mRNAs in patients with amyotrophic lateral sclerosis, leading to degeneration and death of motor neurons controlling voluntary muscle movement [69, 70]. The *ENC1* gene, 490 kb away, encodes Ectoderm-neural cortex protein 1, an actin-binding protein required for adipocyte differentiation [71]

***HCP5 (rs3094013)*:** The lead SNP showed an association with WHR_adjBMI_ in men only (Table 1). The rs3094013 SNP is located in the MHC complex on chromosome 6, and the region within 500kb contains 124 genes (S3 Fig). The known WHR_adjBMI_-increasing allele rs3099844, in strong LD with our lead SNP (r^2^≥0.8), has previously been associated with increased HDL-cholesterol levels [72]. Candidate gene studies suggest that rs1800629 in *tumor necrosis factor* (*TNF*), which is 109 kb upstream and in moderate LD (r^2^ = 0.64) with the lead SNP, may interact with physical activity to decrease serum CRP levels [73, 74]. We did not, however, find an interaction between rs1800629 and physical activity on WHR_adjBMI_ (P = 0.3).

***BAZ1B (rs6976930)*:** There are 31 genes within 500kb the lead SNP rs6976930 (S3 Fig) which is in high LD (r^2^>0.8) with GWAS hits associated with protein C levels, triglycerides, serum urate levels, lipid metabolism, metabolic syndrome, and gamma-glutamyl transferase levels (S14 Table). The rs6976930 SNP shows an eQTL association with *MLXIPL* expression in omental (P = 7x10^-22^) and subcutaneous adipose tissue (P = 4x10^-14^). *MLXIPL* is 122 kb downstream of rs6976930 and codes for a transcription factor that binds carbohydrate response motifs, increasing transcription of genes involved in glycolysis, lipogenesis, and triglyceride synthesis [75, 76].

***PLCE1 (rs10786152)*:** There are 8 genes within 500 kb of the lead SNP (S3 Fig). The lead SNP lies within the intron of *PLCE1* encoding a phospholipase involved in cellular growth and differentiation and gene expression among many other biological processes involving phospholipids [77]. Variants in this gene have been shown to cause nephrotic syndrome, type 3 [78]. Nearby variants rs9663362 and rs932764 (r^2^ = 1.0 and 0.85, respectively) have been previously associated with systolic and diastolic blood pressure (S14 Table).

***CTRB2 (rs889512)*:** The lead SNP showed an association with WHR_adjBMI_ in women only (Table 1). There are 17 genes within 500 kb (S3 Fig). The nearby rs4888378 SNP has been associated with carotid intima-media thickness in women but not in men, and *BCAR1* (*breast cancer anti-estrogen resistance protein 1*) has been implicated as the causal gene [79]. The rs488378 SNP is not, however, in LD with our lead SNP (r^2^<0.1). The SNP rs7202877, in moderate LD with rs889512 (r^2^ = 0.6), is a risk variant for type 1 diabetes (S14 Table). The data from Roadmap Epigenomics Consortium [22] suggest that five variants in strong LD (r^2^>0.8) with our lead SNP rest in known regulatory regions, including rs9936550 within an active enhancer region and rs72802352 in a DNAse hypersensitive region for human skeletal muscle cells and myoblasts; and rs147630228 and rs111869668 within active enhancer regions for the pancreas. Additionally, rs111869668 rests within binding motifs for CEBPB and CEBPD (CCAAT enhancer-binding protein-Beta and Delta) which are enhancer proteins involved in adipogenesis [80, 81].

### Identification of secondary signals

In addition to uncovering 11 novel adiposity loci, our PA-adjusted GWAS and the joint test of SNP main effect and SNP×PA interaction confirmed 148 genome-wide significant loci (50 for BMI, 58 for WC_adjBMI_, 40 for WHR_adjBMI_) that have been established in previous main effect GWAS for adiposity traits (S7–S12 Tables, S4 Fig). The lead SNPs in eight of the previously established loci (5 for BMI, 3 for WC_adjBMI_), however, showed no LD or only weak LD (r^2^<0.3) with the published lead SNP, suggesting they could represent novel secondary signals in known loci (S17 Table). To test whether these eight signals are independent of the previously published signals, we performed conditional analyses [19]. Three of the eight SNPs we examined, in/near *NDUFS4*, *MEF2C-AS1* and *CPA1*, were associated with WC_adjBMI_ with P<5x10^-8^ in our PA-adjusted GWAS even after conditioning on the published lead SNP, hence representing novel secondary signals in these loci (S17 Table).

### Enrichment of the identified loci with functional regulatory elements

Epigenetic variation may underlie gene-environment interactions observed in epidemiological studies [20] and PA has been shown to induce marked epigenetic changes in the genome [21]. We examined whether the BMI or WHR_adjBMI_ loci reaching P<1x10^-5^ for interaction with PA (13 loci for BMI, 5 for WHR_adjBMI_) show overall enrichment with chromatin states in adipose, brain and muscle tissues available from the Roadmap Epigenomics Consortium [22]. However, we did not find significant enrichment (S18 and S19 Tables), which may be due to the limited number of identified loci. The lack of significant findings may also be due to the assessment of chromatin states in the basal state, which may not reflect the dynamic changes that occur when cells are perturbed by PA [23].

We also tested whether the loci reaching P<5x10^-8^ in our PA-adjusted GWAS of BMI or WHR_adjBMI_ show enrichment with chromatin states and found significant enrichment of the BMI loci with enhancer, weak transcription, and polycomb-repressive elements in several brain cell lines, and with enhancer elements in three muscle cell lines (S20 and S21 Tables). We also found significant enrichment of the WHR_adjBMI_ loci with enhancer elements in three adipose and six muscle cell lines, with active transcription start sites in two adipose cell lines, and with polycomb-repressive elements in seven brain cell lines. The enrichment of our PA-adjusted main effect results with chromatin annotations in skeletal muscle in particular, the tissue most affected by PA, could highlight regulatory mechanisms that may be influenced by PA.

## Discussion

In this genome-wide meta-analysis of more than 200,000 adults, we do not find evidence of interaction with PA for loci other than the established *FTO* locus. However, when adjusting for PA or jointly testing for SNP main effect and interaction with PA, we identify 11 novel adiposity loci, suggesting that accounting for PA or other environmental factors that contribute to variation in adiposity may increase power for gene discovery.

Our results suggest that if SNP×PA interaction effects for common variants exist, they are unlikely to be of greater magnitude than observed for *FTO*, the BMI-increasing effect of which is attenuated by ~30% in physically active individuals. The fact that common SNPs explain less of the BMI variance among physically active compared to inactive individuals indicates that further interactions may exist, but larger meta-analyses, more accurate and precise measurement of PA, and/or improved analytical methods will be required to identify them. We found no difference between inactive and active individuals in variance explained by common SNPs in aggregate for WC_adjBMI_ or WHR_adjBMI_, and no loci interacted with PA on WC_adjBMI_ or WHR_adjBMI_. Therefore, PA may not modify genetic influences as strongly for body fat distribution as for overall adiposity. Furthermore, while differences in variance explained by common variants may be due to genetic effects being modified by PA, it is important to note that heritability can change in the absence of changes in genetic effects, if environmental variation differs between the inactive and active groups. Therefore, the lower BMI variance explained in the active group could be partly due to a potentially greater environmental variation in this group.

While we replicated the previously observed interaction between *FTO* and PA [7], it remains unclear what biological mechanisms underlie the attenuation in *FTO*’s effect in physically active individuals, and whether the interaction is due to PA or due to confounding by other environmental exposures. While some studies suggest that *FTO* may interact with diet [24–26], a recent meta-analysis of 177,330 individuals did not find interaction between *FTO* and dietary intakes of total energy, protein, carbohydrate or fat [27]. The obesity-associated *FTO* variants are located in a super-enhancer region [28] and have been associated with DNA methylation levels [29–31], suggesting that this region may be sensitive to epigenetic effects that could mediate the interaction between *FTO* and PA.

In genome-wide analyses for SNP main effects adjusting for PA, or when testing for the joint significance of SNP main effect and SNPxPA interaction, we identify 11 novel adiposity loci, even though our sample size was up to 40% smaller than in the largest published main effect meta-analyses [17, 18]. Our findings suggest that accounting for PA may facilitate the discovery of novel adiposity loci. Similarly, accounting for other environmental factors that contribute to variation in adiposity could lead to the discovery of additional loci.

In the present meta-analyses, statistical power to identify SNPxPA interactions may have been limited due to challenges relating to the measurement and statistical modeling of PA [5]. Of the 60 participating studies, 56 assessed PA by self-report while 4 used wearable PA monitors. Measurement error and bias inherent in self-report estimates of PA [32] can attenuate effect sizes for SNP×PA interaction effects towards the null [33]. Measurement using PA monitors provides more consistent results, but the monitors are not able to cover all types of activities and the measurement covers a limited time span compared to questionnaires [34]. As sample size requirements increase nonlinearly when effect sizes decrease, any factor that leads to a deflation in the observed interaction effect estimates may make their detection very difficult, even when very large population samples are available for analysis. Finally, because of the wide differences in PA assessment tools used among the participating studies, we treated PA as a dichotomous variable, harmonizing PA into inactive and active individuals. Considerable loss of power is anticipated when a continuous PA variable is dichotomized [35]. Our power could be enhanced by using a continuous PA variable if a few larger studies with equivalent, quantitative PA measurements were available.

In summary, while our results suggest that adjusting for PA or other environmental factors that contribute to variation in adiposity may increase power for gene discovery, we do not find evidence of SNP×PA interaction effects stronger than that observed for *FTO*. While other SNP×PA interaction effects on adiposity are likely to exist, combining many small studies with varying characteristics and PA assessment tools may be inefficient for identifying such effects [5]. Access to large cohorts with quantitative, equivalent PA variables, measured with relatively high accuracy and precision, may be necessary to uncover novel SNP×PA interactions.

## Methods

### Main analyses

#### Ethics statement

All studies were conducted according to the Declaration of Helsinki. The studies were approved by the local ethical review boards and all study participants provided written informed consent for the collection of samples and subsequent analyses.

#### Outcome traits—BMI, WC_adjBMI_ and WHR_adjBMI_

We examined three anthropometric traits related to overall adiposity (BMI) or body fat distribution (WC_adjBMI_ and WHR_adjBMI_) [36] that were available from a large number of studies. Before the association analyses, we calculated sex-specific residuals by adjusting for age, age^2^, BMI (for WC_adjBMI_ and WHR_adjBMI_ traits only), and other necessary study-specific covariates, such as genotype-derived principal components. Subsequently, we normalized the distributions of sex-specific trait residuals using inverse normal transformation.

#### Physical activity

Physical activity was assessed and quantified in various ways in the participating studies of the meta-analysis (S1 and S6 Tables). Aiming to amass as large a sample size as possible, we harmonized PA by categorizing it into a simple dichotomous variable—physically inactive vs. active—that could be derived in a relatively consistent way in all participating studies, and that would be consistent with previous findings on gene-physical activity interactions and the relationship between activity levels and health outcomes. In studies with categorical PA data, individuals were defined inactive if they reported having a sedentary occupation and being sedentary during transport and leisure-time (<1 h of moderate intensity leisure-time or commuting PA per week). All other individuals were defined physically active. Previous studies in large-scale individual cohorts have demonstrated that the interaction between *FTO*, or a BMI-increasing genetic risk score, with physical activity, is most pronounced approximately at this activity level [6, 37, 38]. In studies with continuous PA data, PA variables were standardized by defining individuals belonging to the lowest sex- and age-adjusted quintile of PA levels as inactive, and all other individuals as active. The study-specific coding of the dichotomous PA variable in each study is described in S6 Table.

#### Study-specific association analyses

We included 42 studies with genome-wide data, 10 studies with Metabochip data, and eight studies with both genome-wide and Metabochip data. If both genome-wide and Metabochip data were available for the same individual, we only included the genome-wide data (S1 Table). Studies with genome-wide genotyped data used either Affymetrix or Illumina arrays (S2 Table). Following study-specific quality control measures, the genotype data were imputed using the HapMap phase II reference panel (S2 Table). Studies with Metabochip data used the custom Illumina HumanCardio-Metabo BeadChip containing ~195K SNPs designed to support large-scale follow-up of known associations with metabolic and cardiovascular traits [39]. Each study ran autosomal SNP association analyses with BMI, WC_adjBMI_ and WHR_adjBMI_ across their array of genetic data using the following linear regression models in men and women separately: 1) active individuals only; 2) inactive individuals only; and 3) active and inactive individuals combined, adjusting for the PA stratum. In studies that included families or closely related individuals, regression coefficients were estimated using a variance component model that modeled relatedness in men and women combined, with sex as a covariate, in addition to the sex-specific analyses. The additive genetic effect for each SNP and phenotype association was estimated using linear regression. For studies with a case-control design (S1 Table), cases and controls were analyzed separately.

#### Quality control of study-specific association results

All study-specific files for the three regression models listed above were processed through a standardized quality control protocol using the EasyQC software [40]. The study-specific quality control measures included checks on file completeness, range of test statistics, allele frequencies, trait transformation, population stratification, and filtering out of low quality data. Checks on file completeness included screening for missing alleles, effect estimates, allele frequencies, and other missing data. Checks on range of test statistics included screening for invalid statistics such as P-values >1 or <0, negative standard errors, or SNPs with low minor allele count (MAC, calculated as MAF*N, where MAF is the minor allele frequency and N is the sample size) and where SNPs with MAC<5 in the inactive or the active group were removed. The correctness of trait transformation to inverse normal was examined by plotting 2/median of the standard error with the square root of the sample size. Population stratification was examined by calculating the study specific genomic control inflation factor (λ_GC_) [41]. If a study had λ_GC_>1.1, the study analyst was contacted and asked to revise the analyses by adjusting for principal components. The allele frequencies in each study were examined for strand issues and miscoded alleles by plotting effect allele frequencies against the corresponding allele frequencies from the HapMap2 reference panel. Finally, low quality data were filtered out by removing monomorphic SNPs, imputed SNPs with poor imputation quality (r2_hat <0.3 in MACH [42], observed/expected dosage variance <0.3 in BIMBAM [43], proper_info <0.4 in IMPUTE [44]), and genotyped SNPs with a low call-rate (<95%) or that were out of Hardy-Weinberg equilibrium (P<10^−6^).

#### Meta-analyses

Beta-coefficients and standard errors were combined by an inverse-variance weighted fixed effect method, implemented using the METAL software [45]. We performed meta-analyses for each of the three models (active, inactive, active + inactive adjusted for PA) in men only, in women only, and in men and women combined. Study-specific GWAS results were corrected for genomic control using all SNPs. Study-specific Metabochip results as well as the meta-analysis results for GWAS and Metabochip combined were corrected for genomic control using 4,425 SNPs included on the Metabochip for replication of associations with QT-interval, a phenotype not correlated with BMI, WC_adjBMI_ or WHR_adjBMI_, after pruning of SNPs within 500 kb of an anthropometry replication SNP. We excluded SNPs that 1) were not available in at least half of the maximum sample size in each stratum; 2) had a heterogeneity I^2^ >75%, or 3) were missing chromosomal and base position annotation in dbSNP.

#### Calculation of the significance of SNP×PA interaction and of the joint significance of SNP main effect and SNP×PA interaction

To identify SNP×PA interactions, we used the EasyStrata R package [46] to test for the difference in meta-analyzed beta-coefficients between the active and inactive groups for the association of each SNP with BMI, WC_adjBMI_ and WHR_adjBMI_. Easystrata tests for differences in effect estimates between the active and inactive strata by subtracting one beta from the other (β_active_−β_inactive_,) and dividing by the overall standard error of the difference as follows:

$$Z_{diff}=\frac{\beta_{active}-\beta_{inactive}}{\sqrt{\;SE_{active}^{2}-SE_{inactive}^{2}-2\;r\;*\;SE_{active}^{2}*\;SE_{inactive}^{2}}}$$

where *r* is the Spearman rank correlation coefficient between β_active_ and β_inactive_ for all genome-wide SNPs. The joint significance of the SNP main and SNP×PA interaction effects was estimated using the method by Aschard et al. [16] which is a joint test for genetic main effects and gene-environment interaction effects where gene-environment interaction is calculated as the difference in effect estimates between two exposure strata, accounting for 2 degrees of freedom.

#### Testing for secondary signals

Approximate conditional analyses were conducted using GCTA version 1.24 [19]. In the analyses for SNPs identified in our meta-analyses of European-ancestry individuals only, LD correlations between SNPs were estimated using a reference sample comprised of European-ancestry participants of the Atherosclerosis Risk in Communities (ARIC) study. In the analyses for SNPs identified in our meta-analyses of all ancestries combined, the reference sample comprised 93% of European-ancestry individuals and 6% of African ancestry participants from ARIC, as well as 1% of CHB and JPT samples from the HapMap2 panel, to approximate the ancestry mixture in our all ancestry meta-analyses. To test if our identified SNPs were independent secondary signals that fell within 1 Mbp of a previously established signal, we used the GCTA—cojo-cond command to condition our lead SNPs on each previously established SNP in the same locus.

#### Replication analysis for the *CDH12* locus

The replication analysis for the *CDH12* locus included participants from the EPIC-Norfolk (N_INACTIVE_ = 4,755, N_ACTIVE_ = 11,526) and Fenland studies (N_INACTIVE_ = 1,213, N_ACTIVE_ = 4,817), and from the random subcohort of the EPIC-InterAct Consortium (N_INACTIVE_ = 2,154, N_ACTIVE_ = 6,632). PA stratum-specific estimates of the association of *CDH12* with BMI were assessed and meta-analyzed by fixed effects meta-analyses, and the differences between the PA-strata were determined as described above.

### Examining the influence of BMI, WC_adjBMI_ and WHR_adjBMI_-associated loci on other complex traits and their potential functional roles

#### NHGRI-EBI GWAS catalog lookups

To identify associations of the novel BMI, WC_adjBMI_ or WHR_adjBMI_ loci with other complex traits in published GWAS, we extracted previously reported GWAS associations within 500 kb and r^2^>0.6 with any of the lead SNPs, from the GWAS Catalog of the National Human Genome Research Institute and European Bioinformatics Institute [47] (S14 Table).

#### eQTLs

We examined the *cis*-associations of the novel BMI, WC_adjBMI_ or WHR_adjBMI_ loci with the expression of nearby genes from various tissues by performing a look-up in a library of >100 published expression datasets, as described previously by Zhang et al [48]. In addition, we examined *cis*-associations using gene expression data derived from fasting peripheral whole blood in the Framingham Heart Study [49] (n = 5,206), adjusting for PA, age, age^2^, sex and cohort. For each novel locus, we evaluated the association of all transcripts ±1 Mb from the lead SNP. To minimize the potential for false positives, we only considered associations where our lead SNP or its proxy (r^2^>0.8) was either the peak SNP associated with the expression of a gene transcript in the region, or in strong LD (r^2^>0.8) with the peak SNP.

#### Overlap with functional regulatory elements

We used the Uncovering Enrichment Through Simulation method to combine the genetic association data with the Roadmap Epigenomics Project segmentation data [22]. First, 10,000 sets of random SNPs were selected among HapMap2 SNPs with a MAF >0.05 that matched the original input SNPs based on proximity to a transcription start site and the number of LD partners (r^2^>0.8 in individuals of European ancestry in the 1000 Genomes Project). The LD partners were combined with their original lead SNPs to create 10,000 sets of matched random SNPs and their respective LD partners. These sets were intersected with the 15-state ChromHMM data from the Roadmap Epigenomics Project and resultant co-localizations were collapsed from total SNPs down to loci, which were then used to calculate an empirical P value when comparing the original SNPs to the random sets. We examined the enrichment for all loci reaching P<10^−5^ for SNP×PA interaction combined, and for all loci reaching P<5x10^-8^ in the PA-adjusted SNP main effect model combined. In addition, we examined the variant-specific overlap with regulatory elements for each of the index SNPs of the novel BMI, WC_adjBMI_ and WHR_adjBMI_ loci and variants in strong LD (r^2^>0.8).

#### Estimation of variance explained in inactive and active groups

We compared variance explained for BMI, WC_adjBMI_ and WHR_adjBMI_ between the active and inactive groups using two approaches. First, we used a method previously reported by Kutalik et al [15], and selected subsets of SNPs based on varying P value thresholds (ranging from 5x10^-8^ to 0.05) from the SNP main effect model adjusted for PA. Each subset of SNPs was clumped into independent regions using a physical distance criterion of <500kb, and the most significant lead SNP within the respective region was selected. For each lead SNP, the explained variance was calculated as:

$$r^{2}=\frac{1}{1+\;\frac{N}{{\left(\Phi -1\left(\frac{P}{2}\right)\right)}^{2}}}-\frac{1}{N}$$

in the active and inactive groups separately, where *N* is the sample size and *P* is the P value for SNP main effect in active or inactive strata. Finally, the variance explained by each subset of SNPs in the active and inactive strata was estimated by summing up the variance explained by the SNPs.

Second, we applied the LD Score regression tool developed by Bulik-Sullivan et al [14] to quantify the proportion of inflation due to polygenicity (heritability) rather than confounding (cryptic relatedness or population stratification) using meta-analysis summary results. LD Score regression leverages LD between causal and index variants to distinguish true signals by regressing meta-analysis summary results on an ‘LD Score’, i.e. the cumulative genetic variation that an index SNP tags. To obtain heritability estimates by PA strata, we regressed our summary results from the genome-wide meta-analyses of BMI, WC_adjBMI_ and WHR_adjBMI_, stratified by PA status (active and inactive), on pre-calculated LD Scores available in HapMap3 reference samples of up to 1,061,094 variants with MAF≥1% and N>10^th^ percentile of the total sample size.

## Supporting information

S1 Acknowledgements A full list of acknowledgements. (DOCX)

S1 Fig Interaction between the *CDH12* locus and physical activity on BMI in the discovery genome-wide meta-analysis (n = 134,767), in the independent replication sample (n = 31,097), and in the discovery and replication samples combined. (DOCX)

S2 Fig Quantile-Quantile and Manhattan plots for the genome-wide meta-analysis results of the SNP main effect adjusting for physical activity (SNPadjPA), interaction between SNP and physical activity, and the joint effect of SNP main effect and SNP×PA interaction (Joint2df) in men and women of European-ancestry combined. (DOCX)

S3 Fig Regional association plots for novel BMI, WCadjBMI or WHRadjBMI loci showing either a genome-wide significant SNP main effect when adjusting for physical activity as a covariate, or a genome-wide significant joint effect of physical activity-adjusted SNP main effect and SNP × physical activity interaction. (DOCX)

S4 Fig Heatmap of P values for the physical activity-adjusted SNP main effect model (PadjPA), the joint model (Pjoint), and the SNPxPA interaction model (Pint). (DOCX)

S1 Table Basic study information and description of outcome assessment (BMI, WC, WHR) and Physical activity assessment. (XLSX)

S2 Table Genotyping and imputation platforms of the participating studies. (XLSX)

S3 Table Population characteristics for inactive and active individuals combined in the participating studies. (XLSX)

S4 Table Population characteristics for inactive individuals in the participating studies. (XLSX)

S5 Table Population characteristics for active individuals in the participating studies. (XLSX)

S6 Table Methods used for measuring physical activity and definitions of inactive for studies participating in the meta-analyses. (XLSX)

S7 Table All SNPs that met significance for BMI in the European only analyses for at least one of the approaches tested: interaction, adjusted for physical activity, or jointly accounting for the main and interaction effects. (XLSX)

S8 Table All SNPs that met significance for BMI in the all ancestry analyses for at least one of the approaches tested: interaction, adjusted for physical activity, or jointly accounting for the main and interaction effects. (XLSX)

S9 Table All SNPs that met significance for waist circumference adjusted for BMI in the European only analyses for at least one of the approaches tested: interaction, adjusted for physical activity, or jointly accounting for the main and interaction effects. (XLSX)

S10 Table All SNPs that met significance for waist circumference adjusted for BMI in the all ancestry analyses for at least one of the approaches tested: interaction, adjusted for physical activity, or jointly accounting for the main and interaction effects. (XLSX)

S11 Table All SNPs that met significance for waist-to-hip ratio adjusted for BMI in the European only analyses for at least one of the approaches tested: interaction, adjusted for physical activity, or jointly accounting for the main and interaction effects. (XLSX)

S12 Table All SNPs that met significance for waist-to-hip ratio adjusted for BMI in the all ancestry analyses for at least one of the approaches tested: interaction, adjusted for physical activity, or jointly accounting for the main and interaction effects. (XLSX)

S13 Table Variance explained using P value thresholds. (XLSX)

S14 Table GWAS catalog lookups for novel loci and new secondary signal in known loci. (XLSX)

S15 Table Association of the novel loci with *cis* gene expression (*cis*-eQTL). (XLSX)

S16 Table Association of loci identified for interaction with physical activity, for physical activity-adjusted SNP main effect, or for joint association of SNP main effect and physical activity interaction, with physical activity and sedentary behaviour. (XLSX)

S17 Table Results for approximate conditional analyses to identify secondary signals in the novel BMI, WC_adjBMI_ or WHR_adjBMI_-associated loci^a^. (XLSX)

S18 Table Enrichment of loci interacting with PA (P_int_<10^−5^) on the level of BMI with functional genomic elements in adipose, brain, and muscle tissue cell lines from the Roadmap Epigenomics Project. (XLSX)

S19 Table Enrichment of loci interacting with PA (P_int_<10^−5^) on the level of WHRadjBMI with functional genomic elements in adipose, brain, and muscle tissue cell lines from the Roadmap Epigenomics Project. (XLSX)

S20 Table Enrichment of loci showing association with BMI (P_adjPA_<5x10^-8^) with functional genomic elements in adipose, brain, and muscle tissue cell lines from the Roadmap Epigenomics Project. (XLSX)

S21 Table Enrichment of loci showing association with WHRadjBMI (P_adjPA_<5x10^-8^) with functional genomic elements in adipose, brain, and muscle tissue cell lines from the Roadmap Epigenomics Project. (XLSX)

S22 Table Association of novel loci identified for interaction with physical activity, for physical activity-adjusted SNP main effect, or for joint association of the SNP main effect and physical activity interaction, in GIANT results not accounting for physical activity. (XLSX)
