# Correction: Genome-wide physical activity interactions in adiposity ― A meta-analysis of 200,452 adults

**DOI:** 10.1371/journal.pgen.1006972

**Published:** 2017-08-23

**Authors:** Mariaelisa Graff, Robert A. Scott, Anne E. Justice, Kristin L. Young, Mary F. Feitosa, Llilda Barata, Thomas W. Winkler, Audrey Y. Chu, Anubha Mahajan, David Hadley, Luting Xue, Tsegaselassie Workalemahu, Nancy L. Heard-Costa, Marcel den Hoed, Tarunveer S. Ahluwalia, Qibin Qi, Julius S. Ngwa, Frida Renström, Lydia Quaye, John D. Eicher, James E. Hayes, Marilyn Cornelis, Zoltan Kutalik, Elise Lim, Jian’an Luan, Jennifer E. Huffman, Weihua Zhang, Wei Zhao, Paula J. Griffin, Toomas Haller, Shafqat Ahmad, Pedro M. Marques-Vidal, Stephanie Bien, Loic Yengo, Alexander Teumer, Albert Vernon Smith, Meena Kumari, Marie Neergaard Harder, Johanne Marie Justesen, Marcus E. Kleber, Mette Hollensted, Kurt Lohman, Natalia V. Rivera, John B. Whitfield, Jing Hua Zhao, Heather M. Stringham, Leo-Pekka Lyytikäinen, Charlotte Huppertz, Gonneke Willemsen, Wouter J. Peyrot, Ying Wu, Kati Kristiansson, Ayse Demirkan, Myriam Fornage, Maija Hassinen, Lawrence F. Bielak, Gemma Cadby, Toshiko Tanaka, Reedik Mägi, Peter J. van der Most, Anne U. Jackson, Jennifer L. Bragg-Gresham, Veronique Vitart, Jonathan Marten, Pau Navarro, Claire Bellis, Dorota Pasko, Åsa Johansson, Søren Snitker, Yu-Ching Cheng, Joel Eriksson, Unhee Lim, Mette Aadahl, Linda S. Adair, Najaf Amin, Beverley Balkau, Juha Auvinen, John Beilby, Richard N. Bergman, Sven Bergmann, Alain G. Bertoni, John Blangero, Amélie Bonnefond, Lori L. Bonnycastle, Judith B. Borja, Søren Brage, Fabio Busonero, Steve Buyske, Harry Campbell, Peter S. Chines, Francis S. Collins, Tanguy Corre, George Davey Smith, Graciela E. Delgado, Nicole Dueker, Marcus Dörr, Tapani Ebeling, Gudny Eiriksdottir, Tõnu Esko, Jessica D. Faul, Mao Fu, Kristine Færch, Christian Gieger, Sven Gläser, Jian Gong, Penny Gordon-Larsen, Harald Grallert, Tanja B. Grammer, Niels Grarup, Gerard van Grootheest, Kennet Harald, Nicholas D. Hastie, Aki S. Havulinna, Dena Hernandez, Lucia Hindorff, Lynne J. Hocking, Oddgeir L. Holmens, Christina Holzapfel, Jouke Jan Hottenga, Jie Huang, Tao Huang, Jennie Hui, Cornelia Huth, Nina Hutri-Kähönen, Alan L. James, John-Olov Jansson, Min A. Jhun, Markus Juonala, Leena Kinnunen, Heikki A. Koistinen, Ivana Kolcic, Pirjo Komulainen, Johanna Kuusisto, Kirsti Kvaløy, Mika Kähönen, Timo A. Lakka, Lenore J. Launer, Benjamin Lehne, Cecilia M. Lindgren, Mattias Lorentzon, Robert Luben, Michel Marre, Yuri Milaneschi, Keri L. Monda, Grant W. Montgomery, Marleen H. M. De Moor, Antonella Mulas, Martina Müller-Nurasyid, A. W. Musk, Reija Männikkö, Satu Männistö, Narisu Narisu, Matthias Nauck, Jennifer A. Nettleton, Ilja M. Nolte, Albertine J. Oldehinkel, Matthias Olden, Ken K. Ong, Sandosh Padmanabhan, Lavinia Paternoster, Jeremiah Perez, Markus Perola, Annette Peters, Ulrike Peters, Patricia A. Peyser, Inga Prokopenko, Hannu Puolijoki, Olli T. Raitakari, Tuomo Rankinen, Laura J. Rasmussen-Torvik, Rajesh Rawal, Paul M. Ridker, Lynda M. Rose, Igor Rudan, Cinzia Sarti, Mark A. Sarzynski, Kai Savonen, William R. Scott, Serena Sanna, Alan R. Shuldiner, Steve Sidney, Günther Silbernagel, Blair H. Smith, Jennifer A. Smith, Harold Snieder, Alena Stančáková, Barbara Sternfeld, Amy J. Swift, Tuija Tammelin, Sian-Tsung Tan, Barbara Thorand, Dorothée Thuillier, Liesbeth Vandenput, Henrik Vestergaard, Jana V. van Vliet-Ostaptchouk, Marie-Claude Vohl, Uwe Völker, Gérard Waeber, Mark Walker, Sarah Wild, Andrew Wong, Alan F. Wright, M. Carola Zillikens, Niha Zubair, Christopher A. Haiman, Loic Lemarchand, Ulf Gyllensten, Claes Ohlsson, Albert Hofman, Fernando Rivadeneira, André G. Uitterlinden, Louis Pérusse, James F. Wilson, Caroline Hayward, Ozren Polasek, Francesco Cucca, Kristian Hveem, Catharina A. Hartman, Anke Tönjes, Stefania Bandinelli, Lyle J. Palmer, Sharon L. R. Kardia, Rainer Rauramaa, Thorkild I. A. Sørensen, Jaakko Tuomilehto, Veikko Salomaa, Brenda W. J. H. Penninx, Eco J. C. de Geus, Dorret I. Boomsma, Terho Lehtimäki, Massimo Mangino, Markku Laakso, Claude Bouchard, Nicholas G. Martin, Diana Kuh, Yongmei Liu, Allan Linneberg, Winfried März, Konstantin Strauch, Mika Kivimäki, Tamara B. Harris, Vilmundur Gudnason, Henry Völzke, Lu Qi, Marjo-Riitta Järvelin, John C. Chambers, Jaspal S. Kooner, Philippe Froguel, Charles Kooperberg, Peter Vollenweider, Göran Hallmans, Torben Hansen, Oluf Pedersen, Andres Metspalu, Nicholas J. Wareham, Claudia Langenberg, David R. Weir, David J. Porteous, Eric Boerwinkle, Daniel I. Chasman, CHARGE Consortium, EPIC-InterAct Consortium, PAGE Consortium, Gonçalo R. Abecasis, Inês Barroso, Mark I. McCarthy, Timothy M. Frayling, Jeffrey R. O’Connell, Cornelia M. van Duijn, Michael Boehnke, Iris M. Heid, Karen L. Mohlke, David P. Strachan, Caroline S. Fox, Ching-Ti Liu, Joel N. Hirschhorn, Robert J. Klein, Andrew D. Johnson, Ingrid B. Borecki, Paul W. Franks, Kari E. North, L. Adrienne Cupples, Ruth J. F. Loos, Tuomas O. Kilpeläinen

There are errors in some of the gene names in S7, S8, S9, S10, S11 and S12 Tables. Please view the correct tables below, with the locus numbers removed since they are not defined across all the models in the paper.

## Supporting information

S7 Table All SNPs that met significance for BMI in the European only analyses for at least one of the approaches tested: Interaction, adjusted for physical activity, or jointly accounting for the main and interaction effects. (XLSX)

S8 Table All SNPs that met significance for BMI in the all ancestry analyses for at least one of the approaches tested: Interaction, adjusted for physical activity, or jointly accounting for the main and interaction effects. (XLSX)

S9 Table All SNPs that met significance for waist circumference adjusted for BMI in the European only analyses for at least one of the approaches tested: Interaction, adjusted for physical activity, or jointly accounting for the main and interaction effects. (XLSX)

S10 Table All SNPs that met significance for waist circumference adjusted for BMI in the all ancestry analyses for at least one of the approaches tested: Interaction, adjusted for physical activity, or jointly accounting for the main and interaction effects. (XLSX)

S11 Table All SNPs that met significance for waist-to-hip ratio adjusted for BMI in the European only analyses for at least one of the approaches tested: Interaction, adjusted for physical activity, or jointly accounting for the main and interaction effects. (XLSX)

S12 Table All SNPs that met significance for waist-to-hip ratio adjusted for BMI in the all ancestry analyses for at least one of the approaches tested: Interaction, adjusted for physical activity, or jointly accounting for the main and interaction effects. (XLSX)
