# Supplementary material for: Genome-wide physical activity interactions in adiposity ― A meta-analysis of 200,452 adults
Source: PLoS Genet. 2017 Apr 27;13(4):e1006528. doi: 10.1371/journal.pgen.1006528 (PMC5407576; doi:10.1371/journal.pgen.1006528)
Supplement: S1 Acknowledgements — (DOCX) [file pgen.1006528.s001.docx]

**Members of the PAGE Consortium**

Active PAGE investigators who contributed to the present analyses included:

***Coordinating Center***: Rutgers University, Piscataway, NJ: Steve Buyske

***NHGRI***: Division of Genomic Medicine, NHGRI, NIH, Bethesda, MD: Lucia Hindorff.

***MEC***: University of Hawaii, Honolulu, HI: Loïc Le Marchand, Unhee Lim; Keck School of Medicine, University of Southern California, Los Angeles, CA: Christopher Haiman

***WHI***: Fred Hutchinson Cancer Research Institute (FHCRC), Seattle, WA: Charles Kooperberg, Ulrike Peters, Jian Gong, Stephanie Bien

**Acknowledgements**

**AGES:** The Reykjavik Study cohort originally comprised a random sample of 30,795 men and women born in 1907-1935 and living in Reykjavik in 1967. A total of 19,381 attended, resulting in 71% recruitment rate. Between 2002 and 2006, the AGES-Reykjavik study re-examined 5,764 survivors of the original cohort who had participated before in the Reykjavik Study. [Harris, T. B. et al. (2007). American Journal of Epidemiology, 165(9), 1076-1087. doi:10.1093/aje/kwk115]. This study has been funded by NIH contract N01-AG-1-2100, the NIA Intramural Research Program, Hjartavernd (the Icelandic Heart Association), and the Althingi (the Icelandic Parliament). The study is approved by the Icelandic National Bioethics Committee, VSN: 00-063. The researchers are indebted to the participants for their willingness to participate in the study.

**ARIC:** The Atherosclerosis Risk in Communities Study is carried out as a collaborative study supported by National Heart, Lung, and Blood Institute contracts N01-HC-55015, N01-HC-55016, N01-C-55018, N01-HC-55019, N01-HC-55020, N01-HC-55021, N01-HC-55022, R01HL087641, R01HL59367 and R01HL086694; National Human Genome Research Institute contract U01HG004402; and National Institutes of Health contract HHSN268200625226C. Infrastructure was partly supported by Grant Number UL1RR025005, a component of the National Institutes of Health and NIH Roadmap for Medical Research. The project described was supported by Grant Number UL1 RR 025005 from the National Center for Research Resources (NCRR), a component of the National Institutes of Health (NIH) and NIH Roadmap for Medical Research, and its contents are solely the responsibility of the authors and do not necessarily represent the official view of NCRR or NIH. The authors thank the staff and participants of the ARIC Study for their important contributions

**AUSTWIN:** We acknowledge the contributions of many staff in the Genetic Epidemiology Unit, Queensland Institute of Medical Research, in interviewing study participants, sample processing and DNA extraction, and data management. Funding for aspects of this work was provided by the Australian National Health and Medical Research Council (241944, 339462, 389927, 389875, 389891, 389892, 389938, 442915, 442981, 496739, 552485, 552498), the EU 5th Framework Programme GenomeEUTwin Project (QLG2-CT-2002-01254), and the U.S. National Institutes of Health (AA07535, AA10248, AA11998, AA13320, AA13321, AA13326, AA14041, AA17688, DA12854, MH66206 ). A portion of the genotyping on which this study was based was carried out at the Center for Inherited Disease Research, Baltimore (CIDR) (Illumina 370K scans on 4300 individuals), through an access award to our late colleague Dr. Richard Todd. Parts of the statistical analyses were carried out on the Genetic Cluster Computer, which is financially supported by the Netherlands Scientific Organization (NWO 480-05-003). R.P.S.M.  was, and G.W.M. is, supported by National Health and Medical Research Council (NHMRC) Fellowship Schemes. The data collection was supported by a grant to NGM from the Australian Associated Brewers.

**BHS:** The Busselton Health Study acknowledges the generous support for the 1994/5 follow-up study from Healthway, Western Australia and the numerous Busselton community volunteers who assisted with data collection and the study participants from the Shire of Busselton. The Busselton Health Study is supported by The Great Wine Estates of the Margaret River region of Western Australia. The BHS gratefully acknowledges the assistance of the Western Australian DNA Bank (NHMRC Enabling Facility) with DNA samples and the support provided by the Ark (NHMRC Enabling Facility) for this study.

**B58C:** We acknowledge use of phenotype and genotype data from the British 1958 Birth Cohort DNA collection, funded by the Medical Research Council grant G0000934 and the Wellcome Trust grant 068545/Z/02. Genotyping for the B58C-WTCCC subset was funded by the Wellcome Trust grant 076113/B/04/Z. The B58C-T1DGC genotyping utilized resources provided by the Type 1 Diabetes Genetics Consortium, a collaborative clinical study sponsored by the National Institute of Diabetes and Digestive and Kidney Diseases (NIDDK), National Institute of Allergy and Infectious Diseases (NIAID), National Human Genome Research Institute (NHGRI), National Institute of Child Health and Human Development (NICHD), and Juvenile Diabetes Research Foundation International (JDRF) and supported by U01 DK062418. B58C-T1DGC GWAS data were deposited by the Diabetes and Inflammation Laboratory, Cambridge Institute for Medical Research (CIMR), University of Cambridge, which is funded by Juvenile Diabetes Research Foundation International, the Wellcome Trust and the National Institute for Health Research Cambridge Biomedical Research Centre; the CIMR is in receipt of a Wellcome Trust Strategic Award (079895). The B58C-GABRIEL genotyping was supported by a contract from the European Commission Framework Programme 6 (018996) and grants from the French Ministry of Research.

**CARDIA:** The Coronary Artery Risk Development in Young Adults Study (CARDIA) is conducted and supported by the National Heart, Lung, and Blood Institute (NHLBI) in collaboration with the University of Alabama at Birmingham (HHSN268201300025C & HHSN268201300026C), Northwestern University (HHSN268201300027C), University of Minnesota (HHSN268201300028C), Kaiser Foundation Research Institute (HHSN268201300029C), and Johns Hopkins University School of Medicine (HHSN268200900041C).  CARDIA is also partially supported by the Intramural Research Program of the National Institute on Aging (NIA) and an intra-agency agreement between NIA and NHLBI (AG0005).  This manuscript has been reviewed by CARDIA for scientific content.

**CHARGE consortium:** We acknowledge the Cohorts for Heart and Aging Research in Genomic Epidemiology (CHARGE) Consortium for encouraging CHARGE studies to participate in this effort and for the contributions of CHARGE members to the analyses conducted for this research.

**CLHNS:** The Cebu Longitudinal Health and Nutrition Survey (CLHNS) was supported by National Institutes of Health grants DK078150, TW05596, HL085144 and TW008288 and pilot funds from RR20649, ES10126, and DK56350. We thank the Office of Population Studies Foundation research and data collection teams and the study participants who generously provided their time for this study.

**COLAUS:** The CoLaus study was and is supported by research grants from GlaxoSmithKline, the Faculty of Biology and Medicine of Lausanne, and the Swiss National Science Foundation (grants 33CSCO-122661, 33CS30-139468 and 33CS30-148401). The authors thank Vincent Mooser and Dawn Waterworth, Co-PIs of the CoLaus study. Special thanks to Yolande Barreau, Mathieu Firmann, Vladimir Mayor, Anne-Lise Bastian, Binasa Ramic, Martine Moranville, Martine Baumer, Marcy Sagette, Jeanne Ecoffey and Sylvie Mermoud for data collection. SB is supported by the Swiss National Science Foundation (grant 3100AO-116323/1) and the Swiss Institute of Bioinformatics. ZK received financial support from the Leenaards Foundation, the Swiss Institute of Bioinformatics and the Swiss National Science Foundation (31003A-143914, 51RTP0_151019).

**CROATIA-Korcula:** We would like to acknowledge the contributions of the recruitment team in Korcula, the administrative teams in Croatia and Edinburgh and the people of Korcula. The SNP genotyping for the CROATIA-Korcula cohort was performed in Helmholtz Zentrum München, Neuherberg, Germany. The study was financed by the Medical Research Council UK, the Ministry of Science, Education and Sport in the Republic of Croatia (grant number 108-1080315-0302) and the Croatian Science Foundation (grant 8875).

**CROATIA-Vis:** We would like to acknowledge the staff of several institutions in Croatia that supported the field work, including but not limited to The University of Split and Zagreb Medical Schools, Institute for Anthropological Research in Zagreb and Croatian Institute for Public Health. The SNP genotyping for the CROATIA-Vis cohort was performed in the core genotyping laboratory of the Wellcome Trust Clinical Research Facility at the Western General Hospital, Edinburgh, Scotland. This study was supported through grants from the Medical Research Council UK, the Ministry of Science, Education and Sport of the Republic of Croatia (number 108-1080315-0302) and the European Union framework program 6 EUROSPAN project (contract no. LSHG-CT-2006-018947).

**DESIR:** This study was supported in part by grants from SFD ("Société Francophone du 358 Diabète"), CPER ("Contrat de Projets État-Région"), and ANR ("Agence Nationale de la 359 Recherche"). The D.E.S.I.R. study has been supported by INSERM contracts with CNAMTS, Lilly, Novartis Pharma and Sanofi-Aventis; by INSERM (Réseaux en Santé Publique, Interactions entre les déterminants de la santé), Cohortes Santé TGIR, the Association Diabète Risque Vasculaire, the Fédération Française de Cardiologie, La Fondation de France, ALFEDIAM, ONIVINS, Ardix Medical, Bayer Diagnostics, Becton Dickinson, Cardionics, Merck Santé, Novo Nordisk, Pierre Fabre, Roche, Topcon.

**DR’s EXTRA:** The DR.s EXTRA Study was supported by grants to R. Rauramaa by the Ministry of Education and Culture of Finland (627;2004-2011), Academy of Finland (102318; 123885), Kuopio University Hospital , Finnish Diabetes Association, Finnish Heart Association, Päivikki and Sakari Sohlberg Foundation and by grants from European Commission FP6 Integrated Project (EXGENESIS); LSHM-CT-2004-005272, City of Kuopio and Social Insurance Institution of Finland (4/26/2010).

**EGCUT:** EGCUT received support from EU FP7 grant BBMRI-LPC (#313010), H2020 grant ePerMed (#692145), targeted financing from Estonian Government IUT20-60, IUT24-6, Estonian Research Roadmap through the Estonian Ministry of Education and Research (3.2.0304.11-0312), Center of Excellence in Genomics (EXCEGEN),  This work was also supported by the US National Institute of Health [R01DK075787].

**Ely:** We are grateful to all the volunteers and to the staff of St. Mary's Street Surgery, Ely and the study team. The Ely Study was funded by the MRC (MC_U106179471) and Diabetes UK. Genotyping in the Ely and Fenland studies was supported in part by an MRC-GlaxoSmithKline pilot programme grant (G0701863).

**EPIC-Norfolk:** The EPIC Norfolk study is supported by programme grants from the Medical Research Council and Cancer Research UK, and by additional support from the European Union, Stroke Association, British Heart Foundation, Research into Ageing, Department of Health, The Wellcome Trust and the Food Standards Agency. Genotyping was in part supported by the MRC-GSK pilot programme grant. We acknowledge the contribution of the staff and participants of the EPIC-Norfolk Study.

**ERF:** The ERF study is funded by the Centre for Medical Systems Biology (CMSB) and Netherlands Consortium for Systems Biology (NCSB), both within the framework of the Netherlands Genomics Initiative (NGI)/Netherlands Organisation for Scientific Research (NWO). ERF study as a part of EUROSPAN (European Special Populations Research Network) was supported by European Commission FP6 STRP grant number 018947 (LSHG-CT-2006-01947) and also received funding from the European Community's Seventh Framework Programme (FP7/2007-2013)/grant agreement HEALTH-F4-2007-201413 by the European Commission under the programme “Quality of Life and Management of the Living Resources” of 5th Framework Programme (no. QLG2-CT-2002-01254) as well as FP7 project EUROHEADPAIN (nr 602633). High-throughput analysis of the ERF data was supported by joint grant from Netherlands Organisation for Scientific Research and the Russian Foundation for Basic Research (NWO-RFBR 047.017.043) and ZonMw grant (2010/31471/ZONMW). We are grateful to all study participants and their relatives, general practitioners and neurologists for their contributions and to P. Veraart for her help in genealogy, J. Vergeer for the supervision of the laboratory work and P. Snijders for his help in data collection.

**FamHS:** This study was supported by grant R01-DK-8925601 from NIDDK and R01HL117078 from NHLBI

**Fenland:** The Fenland Study is funded by the Wellcome Trust and the Medical Research Council (MC_U106179471). We are grateful to all the volunteers for their time and help, and to the General Practitioners and practice staff for assistance with recruitment. We thank the Fenland Study Investigators, Fenland Study Co-ordination team and the Epidemiology Field, Data and Laboratory teams.

**FINRISK (COROGENE CONTROLS):** This study has been funded by the Academy of Finland (grant numbers 139635, 129494, 129322, 250207, 136895 and 263836), the Orion-Farmos Research Foundation, the Finnish Foundation for Cardiovascular Research, and the Sigrid Jusélius Foundation. We are grateful for the THL DNA laboratory for its skillful work to produce the DNA samples used in this study. We thank the Sanger Institute genotyping facilities for genotyping the samples.

**FramHS:** This research was conducted in part using data and resources from the Framingham Heart Study of the National Heart Lung and Blood Institute of the National Institutes of Health and Boston University School of Medicine. The analyses reflect intellectual input and resource development from the Framingham Heart Study investigators participating in the SNP Health Association Resource (SHARe) project. This work was partially supported by the National Heart, Lung and Blood Institute's Framingham Heart Study (Contracts N01-HC-25195 and HHSN268201500001I) and its contract with Affymetrix, Inc for genotyping services (Contract No. N02-HL-6-4278). A portion of this research utilized the Linux Cluster for Genetic Analysis (LinGA-II) funded by the Robert Dawson Evans Endowment of the Department of Medicine at Boston University School of Medicine and Boston Medical Center. This research was partially supported by grant R01-DK089256 from the National Institute of Diabetes and Digestive and Kidney Diseases (MPIs: I.B. Borecki, L.A. Cupples, K. North) and the National Institutes of Health contract no. HHSN268201500001I.

**FUSION:** Support for FUSION was provided by NIH grants R01-DK062370 (to M.B.), R01-DK072193 (to K.L.M.), and intramural project number 1Z01-HG000024 (to F.S.C.). Genome-wide genotyping was conducted by the Johns Hopkins University Genetic Resources Core Facility SNP Center at the Center for Inherited Disease Research (CIDR), with support from CIDR NIH contract no. N01-HG-65403. H.A.K has received funding from the Academy of Finland (support for clinical research careers, grant no 258753).

**Generation Scotland:** We would like to acknowledge the contributions of the families who took part in the Generation Scotland: Scottish Family Health Study, the general practitioners and Scottish School of Primary Care for their help in recruiting them, and the whole Generation Scotland team, which includes academic researchers, IT staff, laboratory technicians, statisticians and research managers. Genotyping was performed at the Wellcome Trust Clinical Research Facility Genetics Core at Western General Hospital, Edinburgh, UK. GS:SFHS is funded by the Scottish Executive Health Department, Chief Scientist Office, grant number CZD/16/6. Exome array genotyping for GS:SFHS was funded by the Medical Research Council UK.

**GENOA:** The Genetic Epidemiology Network of Arteriopathy (GENOA) study is supported by the National Institutes of Health, grant numbers HL054481, HL054457, and HL119443 from National Heart, Lung, Blood Institute. We thank Eric Boerwinkle, PhD from the Human Genetics Center and Institute of Molecular Medicine and Division of Epidemiology, University of Texas Health Science Center, Houston, Texas, USA and Julie Cunningham, PhD from the Department of Health Sciences Research, Mayo Clinic College of Medicine, Rochester, MN, USA for their help with genotyping.

**GIANT consortium:** This work was performed under the auspices of the Genetic Investigation of ANthropometric Traits (GIANT) consortium.

**GLACIER:** The GLACIER study was funded by project grants to Paul W. Franks from Novo Nordisk, the Swedish Heart-Lung Foundation, the Swedish Diabetes Association, Påhlssons Foundation, the Swedish Research Council, Umeå University Career Development Award, and The Heart Foundation of Northern Sweden. Frida Renström was supported by a post-doctoral stipend from the Swedish Heart-Lung Foundation. The investigators thank the staff at the Wellcome Trust Sanger Institute for technical assistance with genotyping. The investigators are indebted to the study participants who dedicated their time and samples to these studies and the staff at the Umeå Medical Biobank and VIP for biomedical data collection and preparation.

**GOOD:** Financial support was received from the Swedish Research Council, the Swedish Foundation for Strategic Research, the ALF/LUA research grant in Gothenburg, the Lundberg Foundation, the Torsten and Ragnar Söderberg's Foundation, the Västra Götaland Foundation, the Göteborg Medical Society, the Novo Nordisk foundation, and the European Commission grant HEALTH-F2-2008-201865-GEFOS. We would like to acknowledge Maria Nethander at the genomics core facility at University of Gothenburg for statistical analyses.

**GOYA:** This study was conducted as part of the activities of the Gene-Diet Interactions in Obesity project (GENDINOB, www.gendinob.dk) and the MRC Integrative Epidemiology Unit (MRC IEU). The data used in the present study is the ADIGEN subset of the GOYA-male cohort that underwent a thorough clinical-physiological examination, supported by the Danish Medical Research Council. We thank all the participants of the study. Tarunveer S. Ahluwalia received his Post Doctoral Fellowship from the GENDINOB project and acknowledges the same. LP and GDS work in a unit supported by the UK Medical Research Council (MC_UU_12013/1 & /4).

**HAPI Old Order Amish:** We gratefully acknowledge our Amish liaisons, field workers and clinic staff and the extraordinary cooperation and support of the Amish community without which these studies would not have been possible. The Amish studies are supported by grants and contracts from the NIH, including U01 HL072515-06, U01 HL84756, Mid-Atlantic Nutrition Obesity Research Center, grant P30 DK72488, and by National Research Initiative Competitive Grant no. 2007-35205-17883 from the USDA National Institute of Food and Agriculture. We thank our Amish research volunteers for their long-standing partnership in research, and the research staff at the Amish Research Clinic for their hard work and dedication. Dr. Fu is funded by AHA Award #10SDG269004; Dr. Cheng is funded by Veterans Affairs Career Development Award (1 IK2 BX001823).

**Health2006:** The Health2006 study was financially supported by grants from the Velux Foundation; the Danish Medical Research Council, Danish Agency for Science, Technology and Innovation; the Aase and Ejner Danielsens foundation; ALK-Abello´ A/S (Hørsholm, Denmark), Timber Merchant Vilhelm Bangs Foundation, MEKOS Laboratories (Denmark) and Research Centre for Prevention and Health, the Capital Region of Denmark. The Health2006 was approved by the Ethical Committee of Copenhagen (KA-20060011) and the Danish Data Protection Agency.

**HPFS:** The study was supported by grants from the National Heart, Lung, and Blood Institute (HL071981, HL034594, HL126024), the National Institute of Diabetes and Digestive and Kidney Diseases (DK091718, DK100383, DK078616), the Boston Obesity Nutrition Research Center (DK46200), and United States – Israel Binational Science Foundation Grant2011036.

**HRS:** HRS is supported by the National Institute on Aging (NIA U01AG009740). The genotyping was funded separately by the National Institute on Aging (RC2 AG036495, RC4 AG039029), and the analysis was funded in part by R03 AG046389. Our genotyping was conducted by the NIH Center for Inherited Disease Research (CIDR) at Johns Hopkins University. Genotyping quality control and final preparation of the data were performed by the Genetics Coordinating Center at the University of Washington.

**HUNT2:** The Nord-Trøndelag Health Study (The HUNT Study) is a collaboration between HUNT Research Centre (Faculty of Medicine, Norwegian University of Science and Technology NTNU), Nord-Trøndelag County Council, Central Norway Health Authority, and the Norwegian Institute of Public Health.

**InCHIANTI:** The InCHIANTI study baseline (1998-2000) was supported as a "targeted project" (ICS110.1/RF97.71) by the Italian Ministry of Health and in part by the U.S. National Institute on Aging (Contracts: 263 MD 9164 and 263 MD 821336).

**Inter99:** This work was supported by research grants from The Novo Nordisk Foundation Center for Basic Metabolic Research, an independent Research Center at the University of Copenhagen that is partially funded by an unrestricted donation from the Novo Nordisk Foundation, The Danish Diabetes Academy and is part of the LuCamp initiative (www.lucamp.org), sponsored by the Lundbeck Foundation. The Inter99 study was funded by the Danish Research Councils, the Health Foundation, Danish Centre for Evaluation and Health Technology Assessment, Copenhagen County, Danish Heart Foundation, Ministry of Health and Prevention, Association of Danish Pharmacies, Augustinus Foundation, Novo Nordisk, Velux Foundation, Becket Foundation, and Ib Henriksens Foundation.

**KORA:** The KORA study was initiated and financed by the Helmholtz Zentrum München – German Research Center for Environmental Health, which is funded by the German Federal Ministry of Education and Research (BMBF) and by the State of Bavaria. Furthermore, KORA research was supported within the Munich Center of Health Sciences (MC-Health), Ludwig-Maximilians-Universität, as part of LMUinnovativ.

**LOLIPOP:** The LOLIPOP study is supported by the National Institute for Health Research (NIHR) Comprehensive Biomedical Research Centre Imperial College Healthcare NHS Trust, the British Heart Foundation (SP/04/002), the Medical Research Council (G0601966,G0700931), the Wellcome Trust (084723/Z/08/Z) the NIHR (RP-PG-0407-10371), European Union FP7 (EpiMigrant, 279143) and Action on Hearing Loss (G51). The work was carried out in part at the NIHR/Wellcome Trust Imperial Clinical Research Facility. We thank the participants and research staff who made the study possible.

**LURIC:** We thank the LURIC study team who were either temporarily or permanently involved in patient recruitment as well as sample and data handling, in addition to the laboratory staff at the Ludwigshafen General Hospital and the Universities of Freiburg and Ulm, Germany. This work was supported by the 7th Framework Program (integrated project AtheroRemo, grant agreement number 201668 and RiskyCAD, grant agreement number 305739) of the European Union and by the INTERREG IV Oberrhein Program (Project A28, Genetic mechanisms of cardiovascular diseases) with support from the European Regional Development Fund (ERDF) and the Wissenschaftsoffensive TMO.

**MEC:** The Multiethnic Cohort study (MEC) characterization of epidemiological architecture is funded through the NHGRI PAGE program (U01HG007397 and its NHGRI ARRA supplement). The MEC study is funded through the National Cancer Institute (U01CA164973).

**MESA:** MESA and the MESA SHARe project are conducted and supported by the National Heart, Lung, and Blood Institute (NHLBI) in collaboration with MESA investigators. Support for MESA is provided by contracts N01-HC-95159, N01-HC-95160, N01-HC-95161, N01-HC-95162, N01-HC-95163, N01-HC-95164, N01-HC-95165, N01-HC-95166, N01-HC-95167, N01-HC-95168, N01-HC-95169, UL1-TR-001079, and UL1-TR-000040. Funding for SHARe genotyping was provided by NHLBI Contract N02-HL-64278.  Genotyping was performed at Affymetrix (Santa Clara, California, USA) and the Broad Institute of Harvard and Massachusetts Institute of Technology (Boston, Massachusetts, USA) using the Affymetrix Genome-Wide Human SNP Array 6.0. Additional support was provided by R01-HL-088451.

**METSIM:** The METSIM study was funded by the Academy of Finland (grants no. 77299 and 124243).

**NESDA:** Funding was obtained from the Netherlands Organization for Scientific Research (Geestkracht program grant 10-000-1002); the Center for Medical Systems Biology (CSMB, NWO Genomics), Biobanking and Biomolecular Resources Research Infrastructure (BBMRI-NL), VU University’s Institutes for Health and Care Research (EMGO+) and Neuroscience Campus Amsterdam, University Medical Center Groningen, Leiden University Medical Center, National Institutes of Health (NIH, R01D0042157-01A, MH081802, Grand Opportunity grants 1RC2 MH089951 and 1RC2 MH089995). Part of the genotyping and analyses were funded by the Genetic Association Information Network (GAIN) of the Foundation for the National Institutes of Health. Computing was supported by BiG Grid, the Dutch e-Science Grid, which is financially supported by NWO.

**NFBC1966:** NFBC1966 received financial support from the Academy of Finland (project grants 104781, 120315, 129269, 1114194, 24300796, Center of Excellence in Complex Disease Genetics and SALVE), University Hospital Oulu, Biocenter, University of Oulu, Finland (75617), NHLBI grant 5R01HL087679-02 through the STAMPEED program (1RL1MH083268-01), NIH/NIMH (5R01MH63706:02), ENGAGE project and grant agreement HEALTH-F4-2007-201413, EU FP7 EurHEALTHAgeing -277849 and the Medical Research Council, UK (G0500539, G0600705, G1002319, PrevMetSyn/SALVE). The DNA extractions, sample quality controls, biobank up-keeping and aliquotting was performed in the National Public Health Institute, Biomedicum Helsinki, Finland and supported financially by the Academy of Finland and Biocentrum Helsinki. We thank the late Professor Paula Rantakallio (launch of NFBC1966), and Ms Outi Tornwall and Ms Minttu Jussila (DNA biobanking). The authors would like to acknowledge the contribution of the late Academician of Science Leena Peltonen.

**NHS:** The study was supported by grants from the National Heart, Lung, and Blood Institute (HL071981, HL034594, HL126024), the National Institute of Diabetes and Digestive and Kidney Diseases (DK091718, DK100383, DK078616), the Boston Obesity Nutrition Research Center (DK46200), and United States – Israel Binational Science Foundation Grant2011036.

**NTR:** This study was funded by the Netherlands Organization for Scientific Research (NWO) and The Netherlands Organisation for Health Research and Development (ZonMW) grants 904-61-090, 985-10-002, 904-61-193,480-04-004, 400-05-717, Addiction-31160008, Middelgroot-911-09-032, Spinozapremie 56-464-14192, Center for Medical Systems Biology (CSMB, NWO Genomics), NBIC/BioAssist/RK(2008.024), Biobanking and Biomolecular Resources Research Infrastructure (BBMRI –NL, 184.021.007). VU University’s Institute for Health and Care Research (EMGO+ ) and Neuroscience Campus Amsterdam (NCA); the European Science Foundation (ESF, EU/QLRT-2001-01254), the European Community's Seventh Framework Program (FP7/2007-2013), ENGAGE (HEALTH-F4-2007-201413); the European Research Council (ERC Advanced, 230374, Starting grant 284 167), Rutgers University Cell and DNA Repository (NIMH U24 MH068457-06), the Avera Institute, Sioux Falls, South Dakota (USA) and the National Institutes of Health (NIH, R01D0042157-01A, MH081802, Grand Opportunity grant 1RC2 MH089951). Part of the genotyping and analyses were funded by the Genetic Association Information Network (GAIN) of the Foundation for the National Institutes of Health.

**NSHD:** This work was funded by the Medical Research Council (MC_UU_12015/3, MC_UU_12015/1), the British Heart Foundation (RG/10/12/28456) and the Wellcome Trust (088869/B/09/Z). We are very grateful to the members of this birth cohort for their continuing interest and participation in the study. We would like to acknowledge the Swallow group, UCL, who performed the DNA extractions.

**NSPHS:** The NSPHS study was funded by the Swedish Medical Research Council (Project Number K2007-66X-20270-01-3) and the Foundation for Strategic Research (SSF). NSPHS as part of EUROSPAN (European Special Populations Research Network) was also supported by European Commission FP6 STRP grant number 01947 (LSHG-CT-2006-01947). This work has also been supported by the Swedish Medical Research Council (Project Number 2011-2354), the Göran Gustafssons Foundation, the Swedish Society for Medical Research (SSMF), the Kjell och Märta Beijers Foundation, The Marcus Borgström Foundation, the Åke Wiberg foundation and the Vleugels Foundation. We are grateful for the contribution of district nurse Svea Hennix for data collection and Inger Jonasson for logistics and coordination of the NSPHS health survey. We would also like to thank all the participants from the community for their interest and willingness to contribute to this study.

**ORCADES:** ORCADES was supported by the Chief Scientist Office of the Scottish Government, the Royal Society, the MRC Human Genetics Unit, Arthritis Research UK and the European Union framework program 6 EUROSPAN project (contract no. LSHG-CT-2006-018947). DNA extractions were performed at the Wellcome Trust Clinical Research Facility in Edinburgh. We would like to acknowledge the invaluable contributions of Lorraine Anderson and the research nurses in Orkney, the administrative team in Edinburgh and the people of Orkney.

**QFS:** The Quebec Family Study was funded by multiple grants from the Medical Research Council of Canada and the Canadian Institutes of Health Research. This work was supported by a team grant from the Canadian Institutes of Health Research (FRN-CCT-83028)

**RISC:** The RISC Study was supported by European Union grant QLG1-CT-2001-01252 and AstraZeneca. David B. Savage acknowledges funding from the Wellcome Trust (grant no 091551). Tim Frayling is supported by the European Research Council grant 323195:GLUCOSEGENES-FP7-IDEAS-ERC.

**Rotterdam Study:** The generation and management of GWAS genotype data for the Rotterdam Study was executed by the Human Genotyping Facility of the Genetic Laboratory of the Department of Internal Medicine, Erasmus MC, Rotterdam, The Netherlands. The GWAS datasets are supported by the Netherlands Organisation of Scientific Research NWO Investments (nr. 175.010.2005.011, 911-03-012), the Genetic Laboratory of the Department of Internal Medicine, Erasmus MC, the Research Institute for Diseases in the Elderly (014-93-015; RIDE2), the Netherlands Genomics Initiative (NGI)/Netherlands Organisation for Scientific Research (NWO) Netherlands Consortium for Healthy Aging (NCHA), project nr. 050-060-810. We thank Pascal Arp, Mila Jhamai, Marijn Verkerk, Lizbeth Herrera and Marjolein Peters, MSc, and Carolina Medina-Gomez, MSc, for their help in creating the GWAS database, and Karol Estrada, PhD, Yurii Aulchenko, PhD, and Carolina Medina-Gomez, MSc, for the creation and analysis of imputed data. The Rotterdam Study is funded by Erasmus Medical Center and Erasmus University, Rotterdam, the Netherlands Organization for the Health Research and Development (ZonMw), the Research Institute for Diseases in the Elderly (RIDE), the Ministry of Education, Culture and Science, the Ministry for Health, Welfare and Sports, the European Commission (DG XII), and the Municipality of Rotterdam. The authors are grateful to the study participants, the staff from the Rotterdam Study and the participating general practitioners and pharmacists.

**SardiNIA:** We thank the many individuals who generously participated in this study. This work was supported by Contract NO1-AG-1–2109 from the National Institute of Ageing, and in part by a grant from the Italian Ministry of Economy and Finance to the CNR for the Project “FaReBio di Qualità” to F Cucca. The efforts of GR Abecasis were supported in part by contract 263-MA-410953 from the NIA to the University of Michigan and by research grant HG002651 and HL084729 from the NIH.

**SHIP:** SHIP is part of the Community Medicine Research net of the University of Greifswald, Germany, which is funded by the Federal Ministry of Education and Research (grants no. 01ZZ9603, 01ZZ0103, and 01ZZ0403), the Ministry of Cultural Affairs as well as the Social Ministry of the Federal State of Mecklenburg-West Pomerania, and the network ‘Greifswald Approach to Individualized Medicine (GANI_MED)’ funded by the Federal Ministry of Education and Research (grant 03IS2061A). Genome-wide data have been supported by the Federal Ministry of Education and Research (grant no. 03ZIK012) and a joint grant from Siemens Healthcare, Erlangen, Germany and the Federal State of Mecklenburg- West Pomerania. The University of Greifswald is a member of the ‘Center of Knowledge Interchange’ program of the Siemens AG and the Caché Campus program of the InterSystems GmbH.

**SORBS:** This work was supported by grants from the German Research Council (SFB- 1052 “Obesity mechanisms”, SPP 1629 TO 718/2-1), from the German Diabetes Association and from the DHFD (Diabetes Hilfs- und Forschungsfonds Deutschland). Inga Prokopenko was funded in part through the European Community's Seventh Framework Programme (FP7/2007-2013), ENGAGE project, grant agreement HEALTH-F4-2007-201413.

**TRAILS:** TRAILS (TRacking Adolescents’ Individual Lives Survey) is a collaborative project involving various departments of the University Medical Center and University of Groningen, the Erasmus University Medical Center Rotterdam, the University of Utrecht, the Radboud Medical Center Nijmegen, and the Parnassia Bavo group, all in the Netherlands. TRAILS has been financially supported by grants from the Netherlands Organization for Scientific Research NWO (Medical Research Council program grant GB-MW 940-38-011; ZonMW Brainpower grant 100-001-004; ZonMw Risk Behavior and Dependence grant 60-60600-97-118; ZonMw Culture and Health grant 261-98-710; Social Sciences Council medium-sized investment grants GB-MaGW 480-01-006 and GB-MaGW 480-07-001; Social Sciences Council project grants GB-MaGW 452-04-314 and GB-MaGW 452-06-004; NWO large-sized investment grant 175.010.2003.005; NWO Longitudinal Survey and Panel Funding 481-08-013); the Dutch Ministry of Justice (WODC), the European Science Foundation (EuroSTRESS project FP-006), Biobanking and Biomolecular Resources Research Infrastructure BBMRI-NL (CP 32), the participating universities, and Accare Center for Child and Adolescent Psychiatry. We are grateful to all adolescents, their parents and teachers who participated in this research and to everyone who worked on this project and made it possible. Statistical analyses were carried out on the Genetic Cluster Computer (http://www.geneticcluster.org), which is financially supported by the Netherlands Scientific Organization (NWO 480-05-003) along with a supplement from the Dutch Brain Foundation.

**TwinsUK:** The study was funded by the Wellcome Trust; European Community’s Seventh Framework Programme (FP7/2007-2013). The study also receives support from the National Institute for Health Research (NIHR) BioResource Clinical Research Facility and Biomedical Research Centre based at Guy's and St Thomas', NHS Foundation Trust and King's College London. Brent Richards is funded by the CIHR, MDEIE, FRSQ and CFI. SNP Genotyping was performed by The Wellcome Trust Sanger Institute and National Eye Institute via NIH/CIDR.

**WGHS:** The WGHS is supported by HL043851 and HL080467 from the National Heart, Lung, and Blood Institute and CA047988 and UM1CA182913 from the National Cancer Institute, the Donald W. Reynolds Foundation and the Fondation Leducq, with collaborative scientific support and funding for genotyping provided by Amgen.

**WHI:** Funding support for the “Epidemiology of putative genetic variants: The Women’s Health Initiative”
study is provided through the NHGRI PAGE program (U01HG007376, HG004790, and its NHGRI ARRA
supplement). The WHI program is funded by the National Heart, Lung, and Blood Institute, National
Institutes of Health, U.S. Department of Health and Human Services through contracts HHSN268201100046C, HHSN268201100001C, HHSN268201100002C, HHSN268201100003C, HHSN268201100004C, and HHSC271201100004C. The authors thank the WHI investigators and staff for their dedication, and the study participants for making the program possible. A full listing of WHI investigators can be found at: http://www.whiscience.org/publications/ WHI_investigators_shortlist.pdf.

**Whitehall:** Dr. Kumari's and Professor Kivimaki's time on this manuscript was partially supported by the National Heart Lung and Blood Institute (NHLBI: HL36310). The Whitehall-II study has been supported by grants from the Medical Research Council (MRC: K013351); British Heart Foundation; Health and Safety Executive; Department of Health; National Institute on Aging (AG13196), US, NIH; Agency for Health Care Policy Research (HS06516); and the John D and Catherine T MacArthur Foundation Research Networks on Successful Midlife Development and Socioeconomic Status and Health.

**YFS:** The Young Finns Study has been financially supported by the Academy of Finland: grants 286284, 134309 (Eye), 126925, 121584, 124282, 129378 (Salve), 117787 (Gendi), and 41071 (Skidi); the Social Insurance Institution of Finland; Competitive State Research Financing of the Expert Responsibility area of Kuopio, Tampere and Turku University Hospitals (grant X51001); Juho Vainio Foundation; Paavo Nurmi Foundation; Finnish Foundation for Cardiovascular Research ; Finnish Cultural Foundation; Tampere Tuberculosis Foundation; Emil Aaltonen Foundation; Yrjö Jahnsson Foundation; Signe and Ane Gyllenberg Foundation; and Diabetes Research Foundation of Finnish Diabetes Association. The expert technical assistance in the statistical analyses by Irina Lisinen is gratefully acknowledged.

**Other acknowledgements:** This work was supported in part through the computational resources and staff expertise provided by the Department of Scientific Computing at the Icahn School of Medicine at Mount Sinai. Mark I McCarthy is a Wellcome Trust Senior Investigator. Inês Barroso was supported by the Wellcome Trust (WT098051). Marcel den Hoed was supported by the Swedish Research Council (2015-03657) and the Swedish Heart-Lung Foundation (20140543). Tuomas O. Kilpeläinen was supported by the Danish Council for Independent Research (DFF – 1333-00124 and Sapere Aude program grant DFF – 1331-00730B). Robert J. Klein was supported by the NHGRI grant U01-HG007033.
