# Supplementary material for: Genome-wide physical activity interactions in adiposity ― A meta-analysis of 200,452 adults
Source: PLoS Genet. 2017 Apr 27;13(4):e1006528. doi: 10.1371/journal.pgen.1006528 (PMC5407576; doi:10.1371/journal.pgen.1006528)

**Supplementary Figure S1**. Interaction between the *CDH12* locus and physical activity on BMI in the discovery genome-wide meta-analysis of BMI (n=134,767), in the independent replication sample (n=31,097), and in the discovery and replication samples combined.


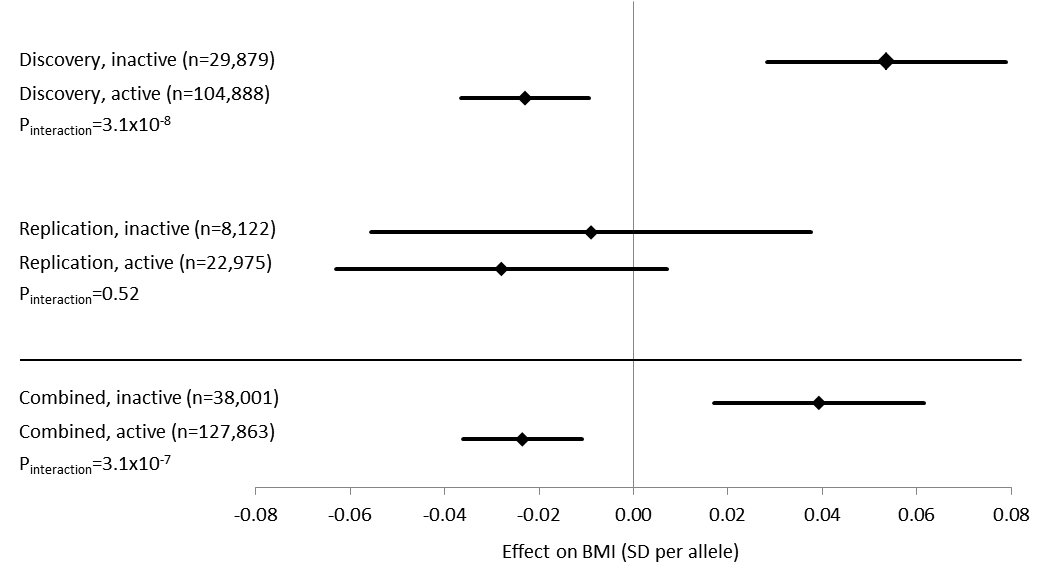

Supplement: S1 Fig — (DOCX) [file pgen.1006528.s002.docx]
