# Supplementary material for: Genome-wide physical activity interactions in adiposity ― A meta-analysis of 200,452 adults
Source: PLoS Genet. 2017 Apr 27;13(4):e1006528. doi: 10.1371/journal.pgen.1006528 (PMC5407576; doi:10.1371/journal.pgen.1006528)
Supplement: S2 Fig — (DOCX) [file pgen.1006528.s003.docx]

**Supplementary Figure S2**. Quantile-Quantile and Manhattan plots for the genome-wide meta-analysis results of the SNP main effect adjusting for physical activity (SNPadjPA), interaction between SNP and physical activity, and the joint effect of SNP main effect and SNPхPA interaction (Joint2df) in men and women of European-ancestry combined. The plots for SNPadjPA and the joint model are shown before and after excluding SNPs ± 500 kb of known main effect loci for BMI, WC_adjBMI_ and WHR_adjBMI_.

**
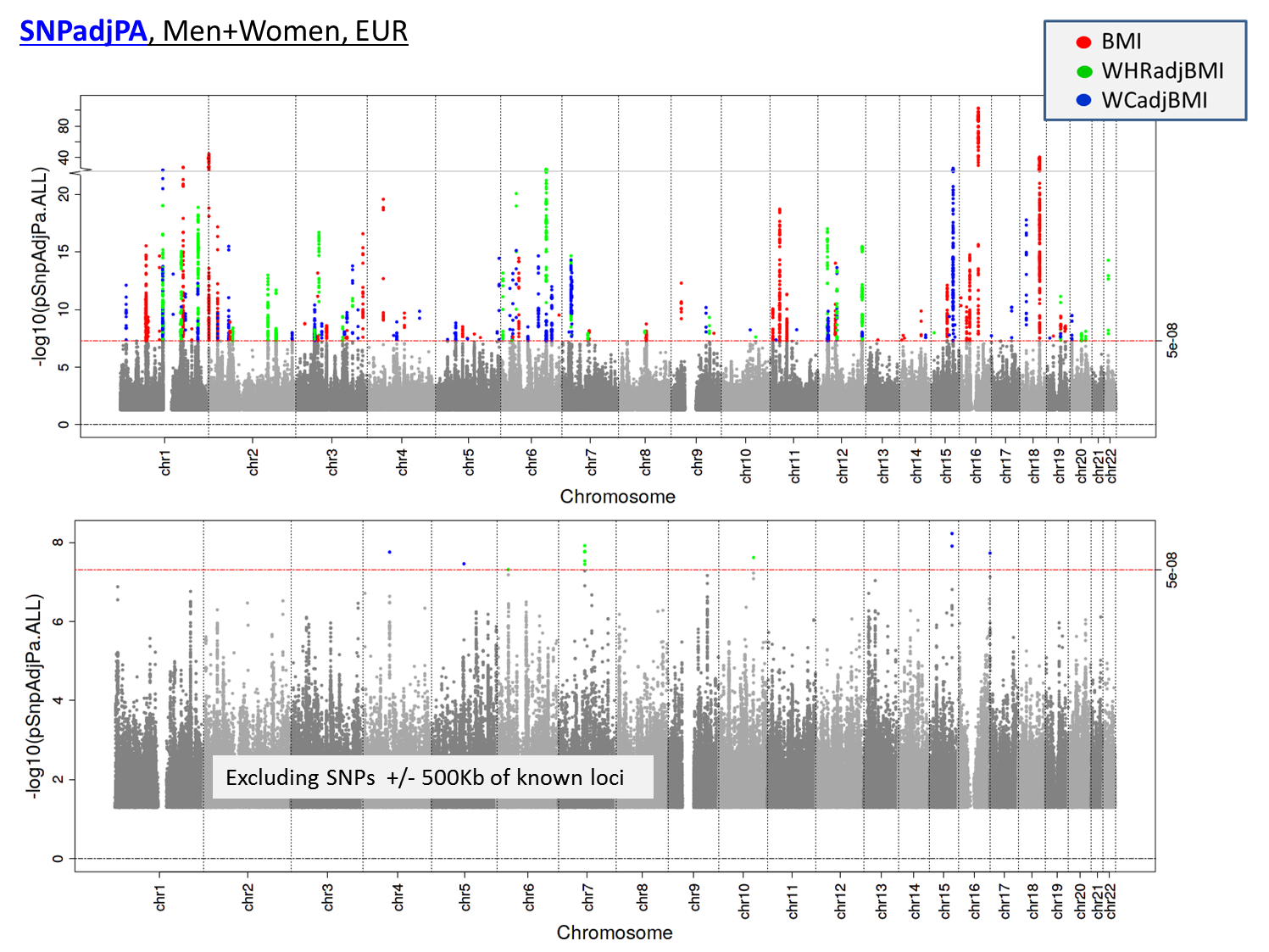

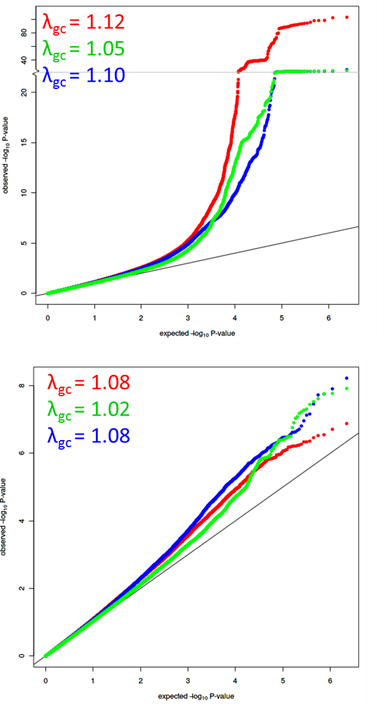
**

**
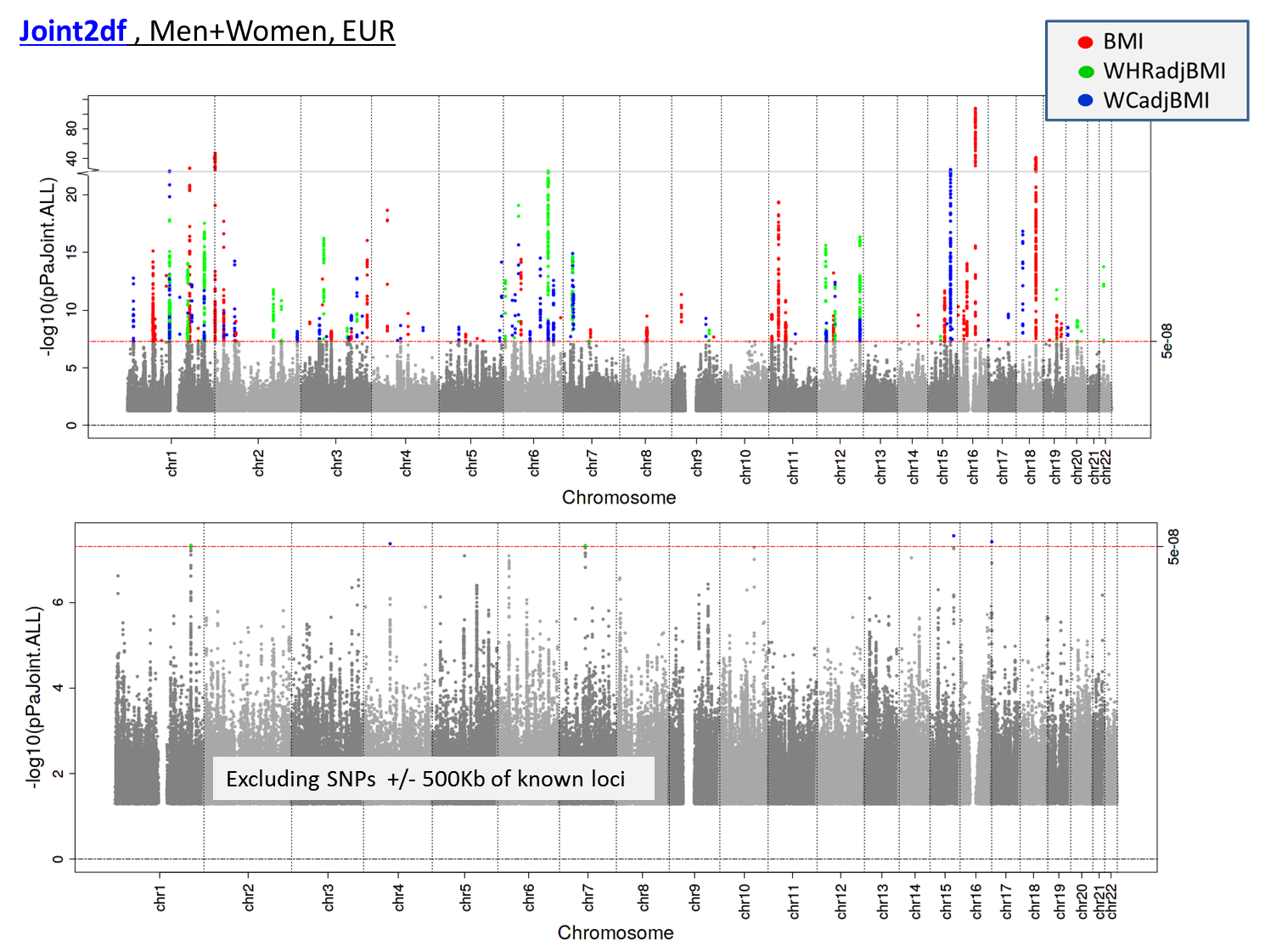

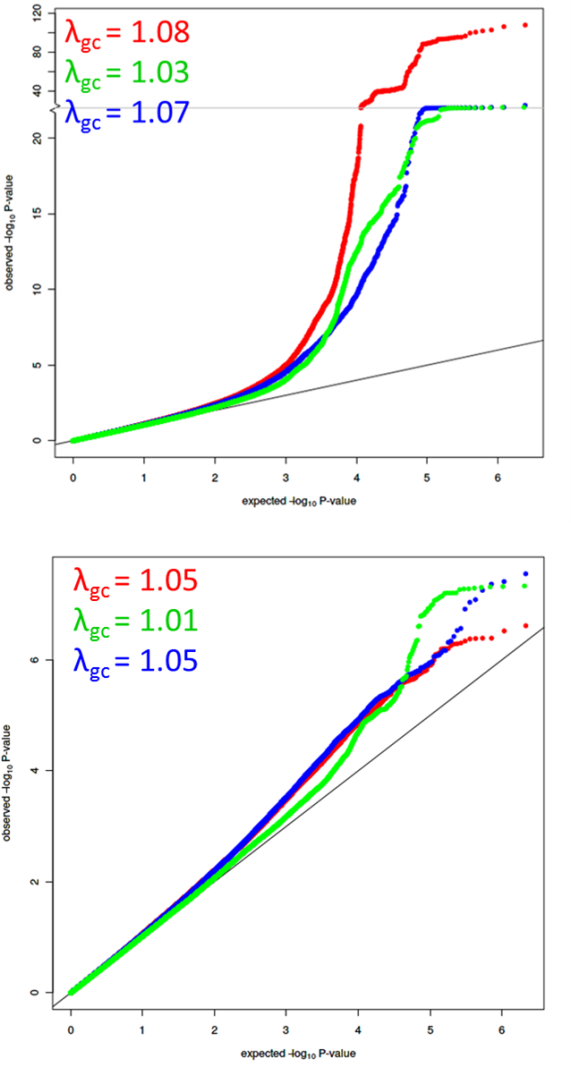
**

**
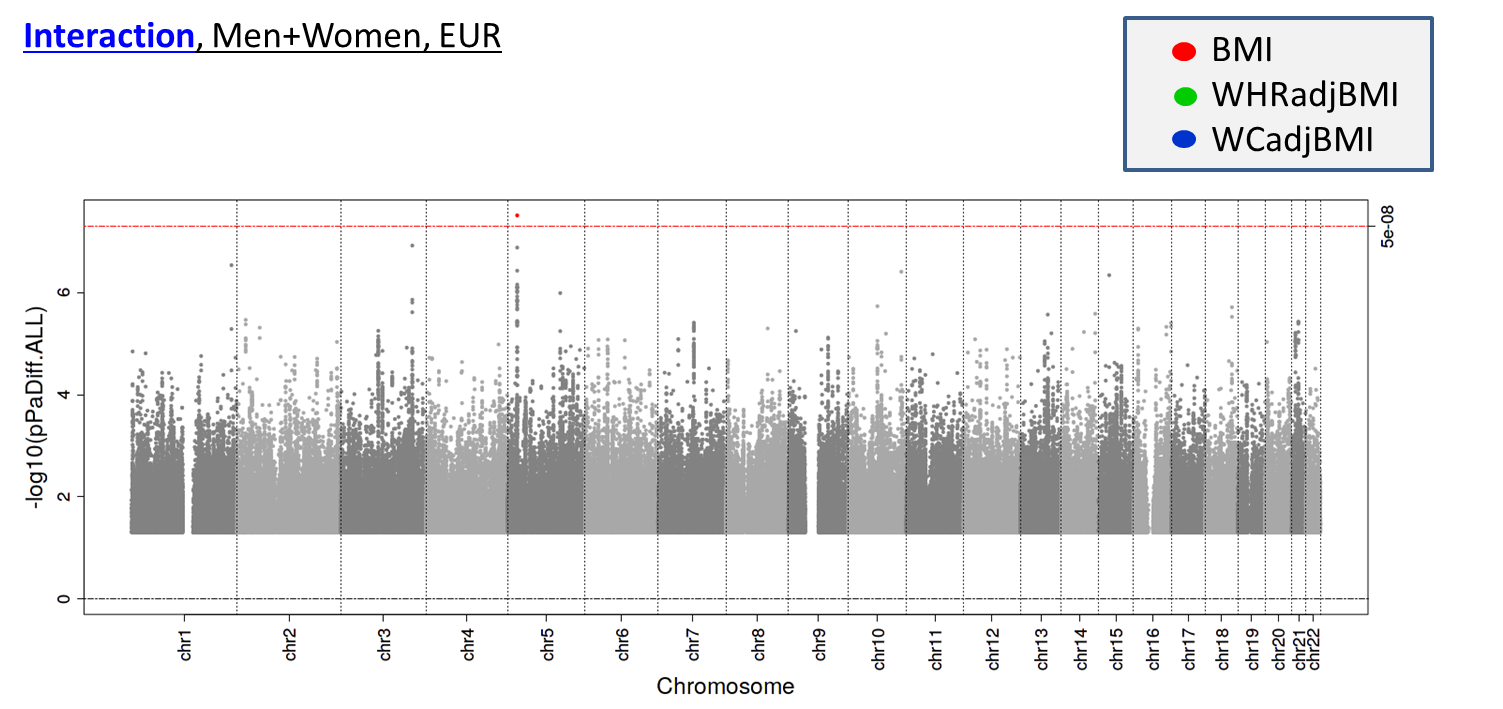

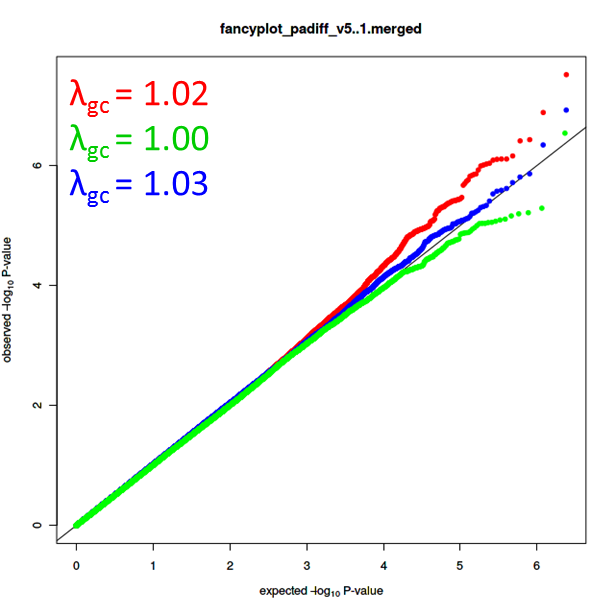
**
