# Supplementary material for: Genome-wide physical activity interactions in adiposity ― A meta-analysis of 200,452 adults
Source: PLoS Genet. 2017 Apr 27;13(4):e1006528. doi: 10.1371/journal.pgen.1006528 (PMC5407576; doi:10.1371/journal.pgen.1006528)
Supplement: S3 Fig — (DOCX) [file pgen.1006528.s004.docx]

**Supplementary Figure S3**. Regional association plots for novel BMI, WC_adjBMI_ or WHR_adjBMI_ loci showing either a genome-wide significant SNP main effect when adjusting for physical activity as a covariate, or a genome-wide significant joint effect of physical activity-adjusted SNP main effect and SNP х physical activity interaction. The plotted results are from meta-analyses of European-ancestry men and women combined, unless otherwise specified.


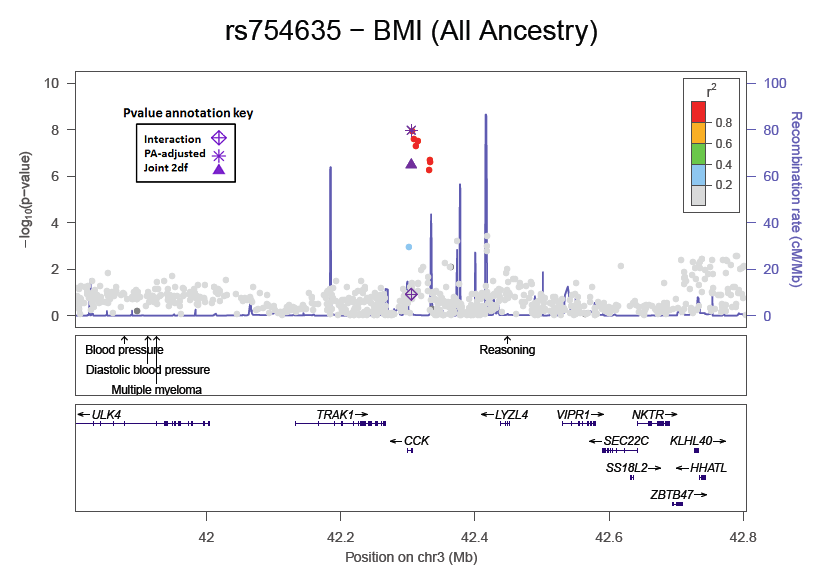

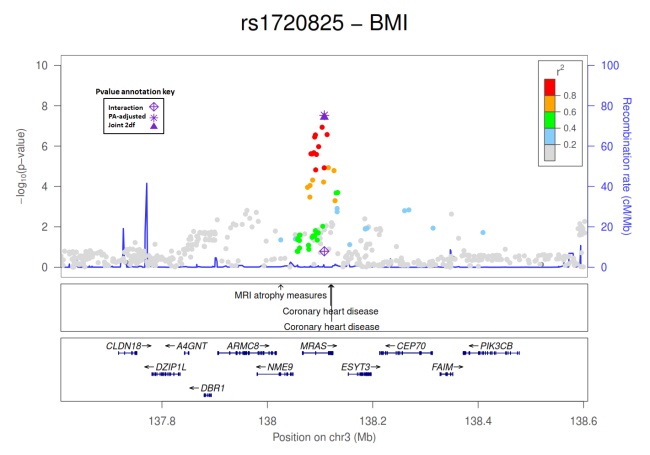

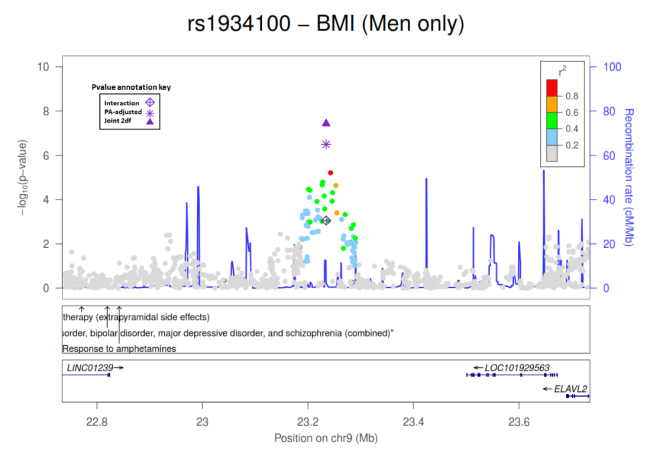

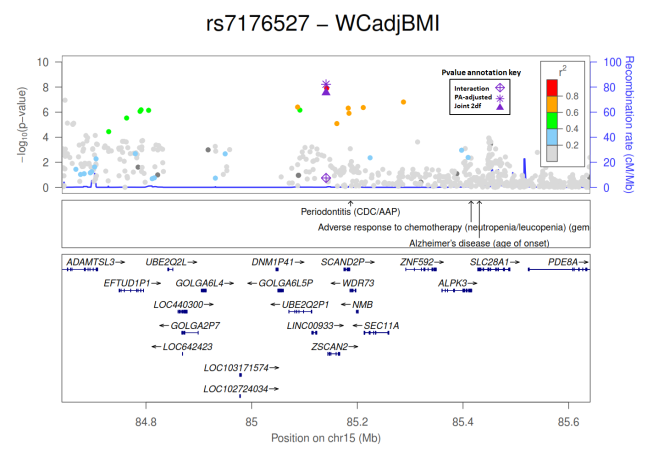
**
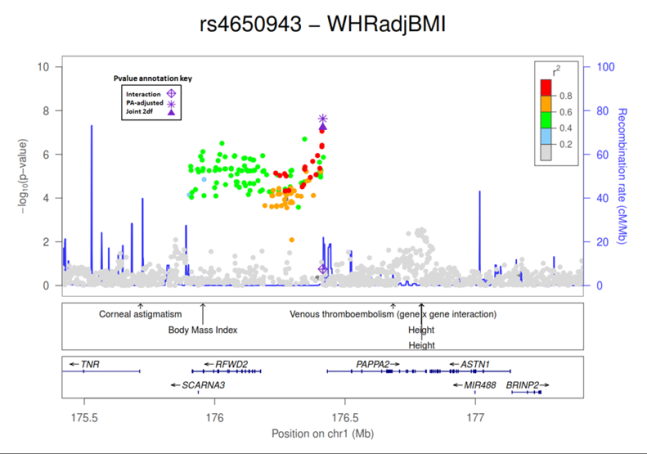
**
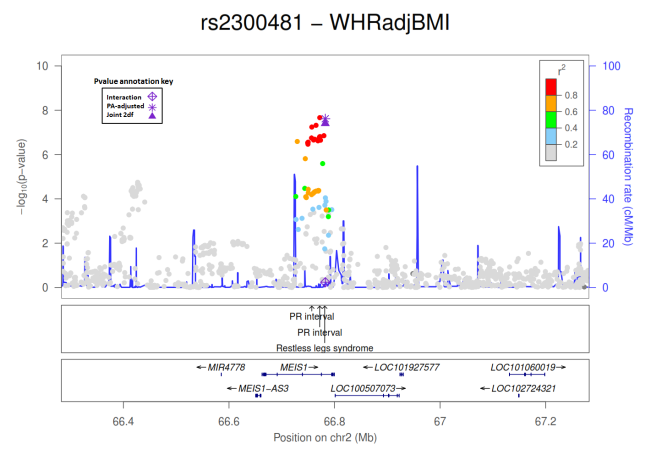


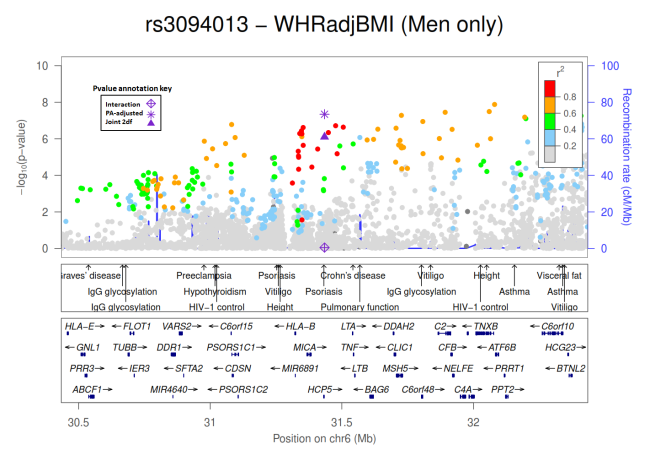

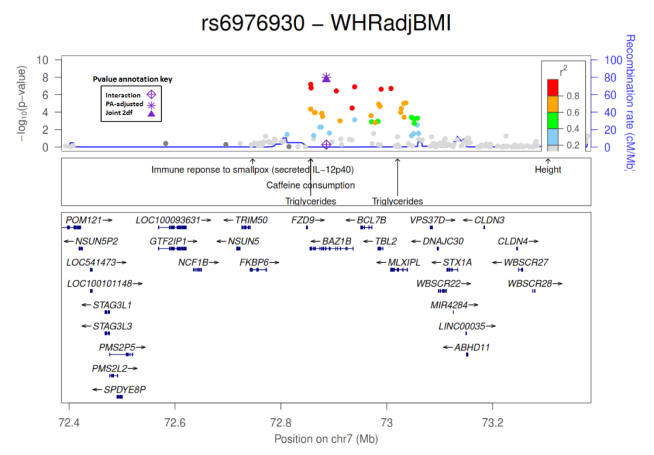
**
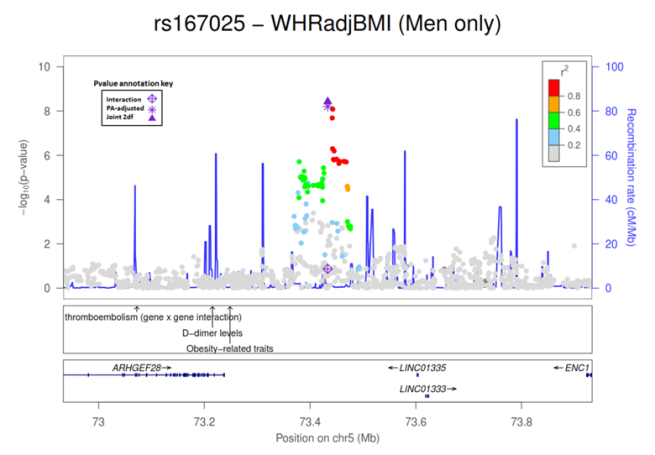


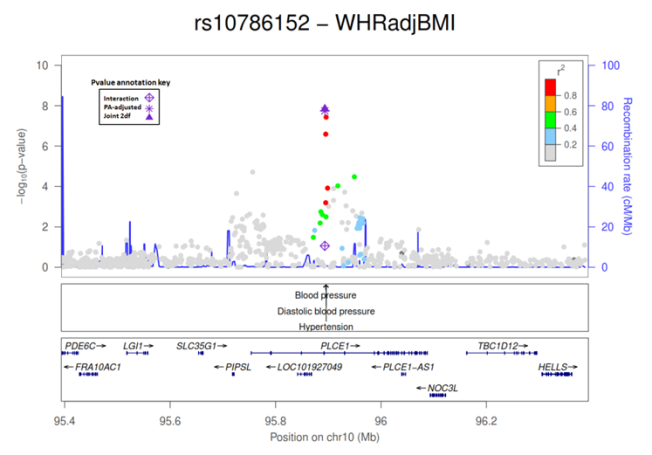

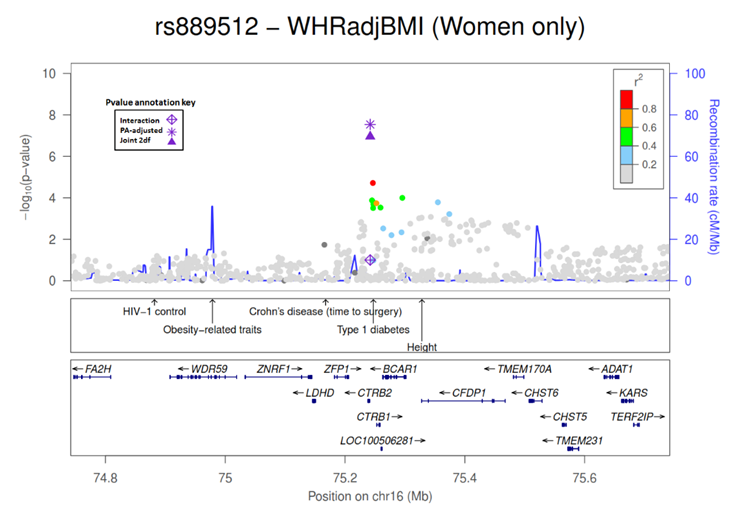
**
