# Supplementary material for: Genome-wide physical activity interactions in adiposity ― A meta-analysis of 200,452 adults
Source: PLoS Genet. 2017 Apr 27;13(4):e1006528. doi: 10.1371/journal.pgen.1006528 (PMC5407576; doi:10.1371/journal.pgen.1006528)
Supplement: S4 Fig — (DOCX) [file pgen.1006528.s005.docx]

**Supplementary Figure S4**. Heatmap of P values for the physical activity-adjusted SNP main effect model (PadjPA), the joint model (Pjoint), and the SNPxPA interaction model (Pint). The figure includes all novel and known variants identified in the present study, and the strength of the color represents the –log10 P-value for association with BMI, WC_adjBMI_, or WHR_adjBMI_ in meta-analyses of European-ancestry only in men and women combined.

**
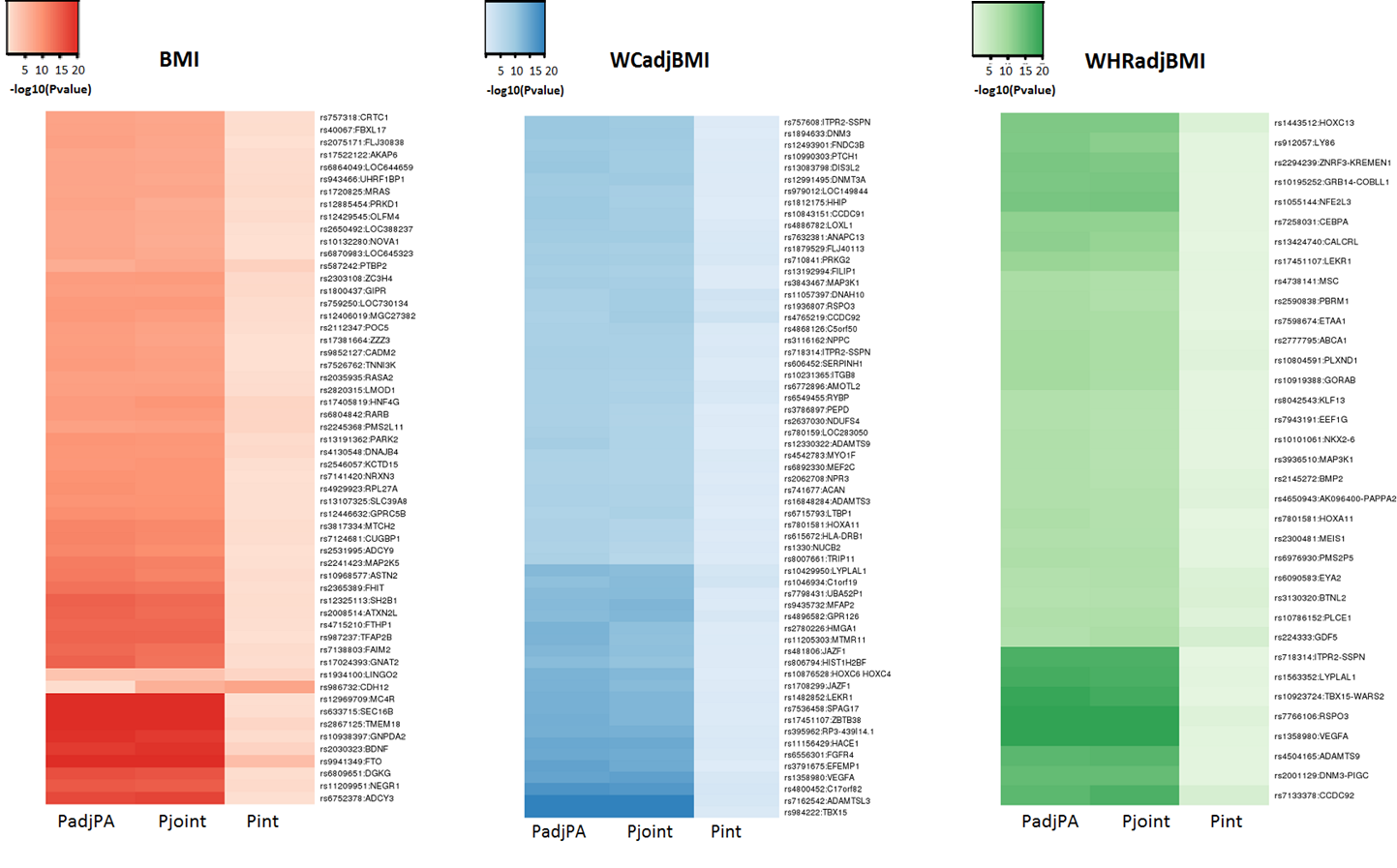
**
